# Performance of SNP barcodes to determine genetic diversity and population structure of *Plasmodium falciparum* in Africa

**DOI:** 10.3389/fgene.2023.1071896

**Published:** 2023-06-01

**Authors:** Dionne C. Argyropoulos, Mun Hua Tan, Courage Adobor, Benedicta Mensah, Frédéric Labbé, Kathryn E. Tiedje, Kwadwo A. Koram, Anita Ghansah, Karen P. Day

**Affiliations:** ^1^ Department of Microbiology and Immunology, Bio21 Institute and Peter Doherty Institute, The University of Melbourne, Melbourne, VIC, Australia; ^2^ Department of Parasitology, Noguchi Memorial Institute for Medical Research, College of Health Sciences, University of Ghana, Accra, Ghana; ^3^ Department of Ecology and Evolution, The University of Chicago, Chicago, IL, United States; ^4^ Epidemiology Department, Noguchi Memorial Institute for Medical Research, University of Ghana, Accra, Ghana

**Keywords:** malaria, high-transmission, molecular surveillance, population genetics, Pf6, single nucleotide polymorphisms, minor allele frequencies, ascertainment bias

## Abstract

Panels of informative biallelic single nucleotide polymorphisms (SNPs) have been proposed to be an economical method to fast-track the population genetic analysis of *Plasmodium falciparum* in malaria-endemic areas. Whilst used successfully in low-transmission areas where infections are monoclonal and highly related, we present the first study to evaluate the performance of these 24- and 96-SNP molecular barcodes in African countries, characterised by moderate-to-high transmission, where multiclonal infections are prevalent. For SNP barcodes it is generally recommended that the SNPs chosen i) are biallelic, ii) have a minor allele frequency greater than 0.10, and iii) are independently segregating, to minimise bias in the analysis of genetic diversity and population structure. Further, to be standardised and used in many population genetic studies, these barcodes should maintain characteristics i) to iii) across various iv) geographies and v) time points. Using haplotypes generated from the MalariaGEN *P. falciparum* Community Project version six database, we investigated the ability of these two barcodes to fulfil these criteria in moderate-to-high transmission African populations in 25 sites across 10 countries. Predominantly clinical infections were analysed, with 52.3% found to be multiclonal, generating high proportions of mixed-allele calls (MACs) per isolate thereby impeding haplotype construction. Of the 24- and 96-SNPs, loci were removed if they were not biallelic and had low minor allele frequencies in all study populations, resulting in 20- and 75-SNP barcodes respectively for downstream population genetics analysis. Both SNP barcodes had low expected heterozygosity estimates in these African settings and consequently biased analyses of similarity. Both minor and major allele frequencies were temporally unstable. These SNP barcodes were also shown to identify weak genetic differentiation across large geographic distances based on Mantel Test and DAPC. These results demonstrate that these SNP barcodes are vulnerable to ascertainment bias and as such cannot be used as a standardised approach for malaria surveillance in moderate-to-high transmission areas in Africa, where the greatest genomic diversity of *P. falciparum* exists at local, regional and country levels.

## 1 Introduction

*Plasmodium falciparum* malaria remains a persistent threat for sub-Saharan Africa, where approximately 95% of total malaria cases and 96% of all malaria deaths occur (World Health Organisation, 2022). With unprecedented rebounds in prevalence since 2016, made worse with the COVID-19 pandemic (World Health Organisation, 2022), elimination targets that have been set to be achieved by 2030 are ambitious. The potential contribution of molecular surveillance to determine changes in population diversity and structure in routine monitoring and evaluation of control and elimination strategies is a topic of active research, with a variety of approaches using putatively neutral variation or antigen-encoding loci being explored.

In the microbiological world, *P. falciparum* presents a special case in the use of these molecular surveillance methods for several reasons. There is a spectrum of population structures from clonal in epidemic settings, to highly diverse in the high burden countries of Africa. This is directly related to transmission intensity (Anderson et al., 2000). With frequent exposure to infected mosquitoes in moderate and high transmission settings, the majority of infections in humans contain multiple distinct *P. falciparum* genomes (ranging from 1 to 20 diverse genomes in a microlitre of blood), which can frequently recombine due to the obligatory sexual (meiotic) phase of the life cycle in the mosquito (Babiker et al., 1994; Paul et al., 1995). Identifying markers that are informative, regardless of recombination intensity, which remain stable across time is challenging, due to the high rate of genetic recombination in *P. falciparum* populations (Escalante et al., 2015). Given the “many epidemiologies of malaria” with associated diverse population structures, the development and performance of molecular surveillance methods need to be evaluated in a range of transmission settings (see (Escalante and Pacheco, 2019) for an extensive review of population genetics in *Plasmodium* spp.). One method may not be the solution for all malaria endemic areas nor for comparative studies.

“Molecular barcodes” of single nucleotide polymorphisms (SNPs) have been proposed as a molecular surveillance tool and heralded as the new frontier of malaria surveillance, revisiting research in human, animal, and plant genetics almost 20 years ago (Syvänen, 2001; Vignal et al., 2002; Ohashi and Tokunaga, 2003; Langridge and Chalmers, 2005). This has been prompted by the needs of scientists in endemic countries for genotyping methods that can be used with standard laboratory equipment, at reasonable costs and without specialised skills. As malaria control and elimination interventions are actioned locally, it is therefore imperative for analyses of genetic diversity and population structure to be performed in-country (Vignal et al., 2002). SNPs are typically biallelic and the benefits of using SNPs include the abundance of annotated markers, low-scoring error rates, transferability of data across laboratories, the ability to genotype neutral and non-neutral regions in the same run, and, in contrast to multiallelic markers such as microsatellites, can largely be fully automated (Khlestkina and Salina, 2006). While microsatellites have been successfully used in moderate-to-high transmission, genotyping these markers are more laborious and cannot be fully automated. Therefore, we wish to evaluate whether SNP barcodes would be useful in these settings.

Small molecular barcodes have been applied to evaluate changes in diversity and population structure of *P. falciparum* as a result of malaria interventions; detect geographic origins of infection, whether local or imported; distinguish parasite clones from one another, using neutral theory; as well as identify spatial differentiation between parasite populations. 24-SNP (Daniels et al., 2008) and 96-SNP (Nkhoma et al., 2013) barcodes have been successfully deployed in low-transmission countries such as those in Southeast Asia (Thailand (Daniels et al., 2008), Thai-Cambodia border (Nkhoma et al., 2013)), South America (Charles et al., 2016), and also in areas of Africa having undergone intense malaria control programmes (Senegal (Daniels et al., 2008; Daniels et al., 2013; Daniels et al., 2015; Bei et al., 2018), Ndirande, Malawi (Sisya et al., 2015) and Madagascar (Rice et al., 2016)). Other genome-wide SNP genotyping panels have been successful to detect intercontinental (Neafsey et al., 2008) and within-country (Aydemir et al., 2018; Tessema et al., 2020; Verity et al., 2020) population structure but require many more SNPs (>500) for the same purpose. However, their utility in highly diverse moderate-to-high transmission settings, where the burden of malaria remains the highest, has not been rigorously assessed.

The immediate problem with the use of SNP barcodes on samples from moderate-to-high transmission settings is the high prevalence of multiclonal infections and whether haplotypes can be accurately constructed for population genetic analysis. This is known as phasing and is more challenging with biallelic SNPs (Chang et al., 2017; Zhu et al., 2018; Gerlovina et al., 2022), compared to more polyallelic microsatellite markers (Anderson et al., 1999). The standard empirical solution in malaria population genetics (Anderson et al., 2000; Tessema et al., 2020) used by the originators of the 24-SNP barcode (Daniels et al., 2008) is to use only single-clone infections with the consequence of drastically reducing the numbers of loci and sample size for analysis. Here we illustrate this point with an analysis of a 24-SNP barcode dataset of asymptomatic infections from a high-transmission malaria endemic region in Obuasi, Ghana (Supplementary Material, ethics approval: CPN 11/04-05). In this dataset, approximately 80% of infections were multiclonal, resulting in a median of 25%–33% of loci with mixed-allele calls (MACs) (i.e., heteroallelic calls) per haplotype. These MACs severely limited the number of isolates available for haplotype construction, necessary to perform population genetics analysis. Motivated by the difficulties in analysing the Obuasi dataset due to the high prevalence of multiclonal infections, we decided to explore further whether this issue was more widespread in other endemic areas in Africa. We tested the suitability of two published SNP barcodes (Daniels et al., 2008; Nkhoma et al., 2013) to identify genetic diversity and population structure in 25 moderate-to-high transmission settings in Africa.

It is recommended that SNP barcodes i) are biallelic, ii) have a minor (least frequent) allele frequency greater than 0.10 and iii) independently segregating, so that genetic diversity and population structure analyses are not biased. Further, for these barcodes to be standardised as a one-size-fits-all panel, they iv) should work across a range of geographies and v) be temporally stable. SNP genotypes of isolates obtained from the MalariaGEN *P. falciparum* community Project version 6 (MalariaGEN et al., 2021) were used to test these criteria in SNP barcodes across African populations. We describe high levels of multiclonal infections and MACs that hindered accurate haplotype construction for population genetics analyses. Nonetheless, there was sufficient data to show haplotype variation with large-scale geographic distance across Africa. Whilst proven to be practical and meaningful in low-transmission settings with a high proportion of monoclonal infections, we suggest that other molecular surveillance methods, not restricted by these limitations, are needed to guide malaria control programmes in endemic settings characterised by moderate-to-high transmission in Africa.

## 2 Methods

### 2.1 MalariaGEN Africa *P. falciparum* dataset

SNP genotypes in African countries were obtained from the MalariaGEN *Plasmodium falciparum* Community Project (version 6, https://www.malariagen.net/resource/26) (MalariaGEN et al., 2021), hereinafter referred to as the “Pf6 dataset”. All samples in the Pf6 dataset were obtained from blood samples from patients with *P. falciparum* malaria with informed consent from the patient or parent/guardian with ethical approval as described in (MalariaGEN et al., 2021). Standard laboratory protocols were used to determine the DNA quantity and proportion of human DNA per sample (Manske et al., 2012; Miles et al., 2016). As *P. falciparum* samples were obtained from human blood samples, the parasite is in its haploid stage.

**FIGURE 1:**
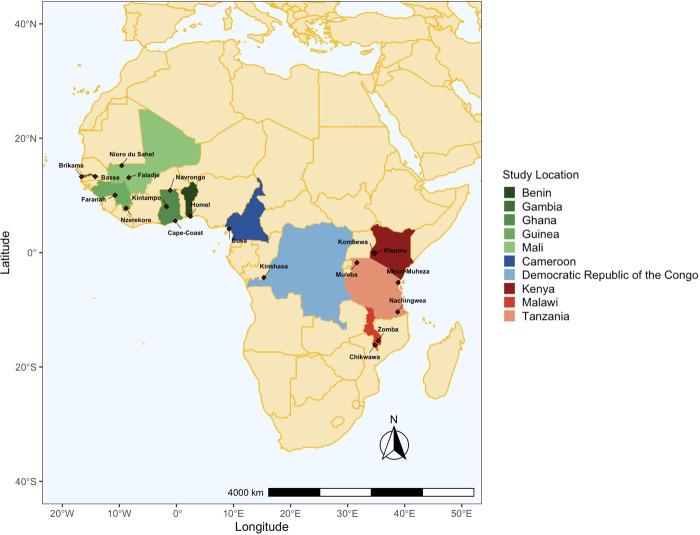
Map of countries and locations in the Pf6 database from Africa included in this study. 2,317 isolates were chosen from locations per year where there was a minimum of 25 isolates (see Methods). Colours indicate the country that isolates were obtained from, and diamonds indicate the specific regions that individuals were sampled with *P. falciparum* infections. The map is segregated into three regions: West Africa (green hues; *n* = 5), Central Africa (blue hues; *n* = 2), and East Africa (red hues; *n* = 3). Latitude/Longitude coordinates for study locations were obtained from the MalariaGEN *Plasmodium falciparum* community Project version 6 (MalariaGEN et al., 2021) isolate study and metadata.

Available metadata included the study ID, country, location and year that each isolate was collected. Isolates were filtered for the following criteria: i) used Whole Genome Sequencing library strategy, ii) passed the quality control (“QC pass”), and iii) sequencing was performed using the Illumina HiSeq 2000 paired-end sequencing platform (MalariaGEN et al., 2021). We used the term “study population” to represent isolates collected from the same location and year. From a total of 2,922 African isolates in the database, study populations that had greater than or equal to 25 isolates and were from study populations defined as moderate- or high-transmission by their respective study and, if not specified, defined by us using the World Health Organisation (WHO/GMP, 2017) were then selected to undergo further analysis (*N* = 2,317 isolates) (Table 1; Supplementary Figure S1). This threshold was used to minimise statistical bias while maximising the number of populations included in the study (Pruett and Winker, 2008; Hoban and Schlarbaum, 2014; Flesch et al., 2018; Qu et al., 2020). These isolates were sampled across 10 countries from 25 study populations in West Africa (Benin, The Gambia, Ghana, Guinea, and Mali), Central Africa (Cameroon and DRC), and East Africa (Kenya, Malawi, and Tanzania) (Figure 1). Supplementary Figure S1 outlines the inclusion/exclusion criteria used to filter isolates and SNP loci to generate final datasets for downstream analyses.

**TABLE 1:** Epidemiological and Study Population Information. Genetic data were obtained for *N* = 2,317 isolates from the Pf6 MalariaGen repository and epidemiological metadata were obtained from study references as indicated in the table.

Region	Country	Study location	Year	Latitude	Longitude	Isolates	Endemicity	Transmission	Malaria disease status	References
West	Benin	Homel	2014	6.3607027	2.4381709	36	Moderate	Double Peak	Clinical	Bertin et al. (2013)
	The Gambia	Basse	2014	13.30944	−14.21925	81	High	Seasonal	Clinical	Amambua-Ngwa et al. (2018)
		Brikama	2014	13.27479	−16.64092	42	Moderate	Seasonal	Clinical	Amambua-Ngwa et al. (2018)
	Ghana	Cape-Coast	2014	5.55602	−0.1969	100	High	Perennial	Clinical	Kamau et al. (2015), Mensah et al. (2020)
		Kintampo	2012	8.0564	−1.72446	35	High	Perennial	Clinical	Mensah-Brown et al. (2015)
		Navrongo	2009	10.885568	−1.086617	46	High	Seasonal	Clinical	MalariaGEN et al. (2021)
		Navrongo	2010	10.885568	−1.086617	135	High	Seasonal	Clinical	MalariaGEN et al. (2021)
		Navrongo	2011	10.885568	−1.086617	93	High	Seasonal	Clinical	MalariaGEN et al. (2021)
		Navrongo	2012	10.885568	−1.086617	39	High	Seasonal	Clinical	Duffy et al. (2015)
		Navrongo	2013	10.885568	−1.086617	241	High	Seasonal	Clinical	Kamau et al. (2015)
		Navrongo	2015	10.885568	−1.086617	57	High	Seasonal	Clinical	MalariaGEN et al. (2021)
	Guinea	Faranah	2011	10.0438	−10.7351	37	High	Perennial	Clinical	Mobegi et al. (2014)
		Nzerekore	2011	7.753857	−8.818703	112	High	Perennial	Clinical	Mobegi et al. (2014)
	Mali	Faladje	2013	13.1333	−8.3333	124	Moderate	Seasonal	Clinical	Kone et al. (2013), Kone et al. (2020), Ghansah et al. (2014), Kamau et al. (2015)
		Nioro du Sahel	2014	15.23199	−9.58863	49	Moderate	Unstable	Clinical	Duffy et al. (2018), Diakité et al. (2019)
Central	Cameroon	Buea	2013	4.14638	9.245531	235	High	Seasonal	Clinical/Asymptomatic	Apinjoh et al. (2015)
	Democratic Republic of Congo (DRC)	Kinshasa	2012	−4.36939	15.320977	171	High	Double Peak	Clinical	Onyamboko et al. (2014)
		Kinshasa	2013	−4.36939	15.320977	108	High	Double Peak	Clinical	Onyamboko et al. (2014)
East	Kenya	Kisumu	2014	−0.0917	34.76796	34	High	Perennial	Clinical	Ngalah et al. (2015), U.S. President’s Malaria Initiative (2015), U.S. President’s Malaria Initiative (2017), Laurent et al. (2018)
		Kombewa	2014	-0.1035	34.5183	26	High	Perennial	Clinical	Ngalah et al. (2015), U.S. President’s Malaria Initiative (2015), U.S. President’s Malaria Initiative (2017), Laurent et al. (2018)
	Malawi	Chikwawa	2011	-16.193575	34.7715	221	High	Perennial	Clinical	Ocholla et al. (2014), Ravenhall et al. (2016)
		Zomba	2011	−15.3891	35.3292	33	High	Perennial	Clinical	U.S. President’s Malaria Initiative (2012), Ravenhall et al. (2016)
	Tanzania	Mkuzi-Muheza	2013	−5.241083	38.82872	145	High	Seasonal	Clinical	Baraka et al. (2015)
		Muleba	2013	−1.750317	31.61992	52	Moderate	Double Peak	Clinical	West et al. (2013), Baraka et al. (2015)
		Nachingwea	2013	−10.36795	38.75465	65	High	Seasonal	Clinical	Baraka et al. (2015)

### 2.2 Description of SNP barcodes

To be able to understand the genetic diversity and population structure of each parasite isolate and test whether small panels or “barcodes” provide enough information, we chose to analyse published 24- and 96-SNP barcodes (Daniels et al., 2008; Nkhoma et al., 2013) that were found to successfully work in low-transmission settings.

#### 2.2.1 24-SNP barcode

We mined each parasite isolate genome for their genotype at the 24 genome-wide SNPs in the “molecular barcode” Taq-Man assay as described (Daniels et al., 2008). Briefly, Daniels et al. (2008) first genotyped over 2,100 SNPs that were discovered through comparative genome sequencing (Volkman et al., 2007) before developing a panel of 24-SNPs that were found to be biallelic, with a high minor allele frequency (MAF >0.35), and had a conserved region around the SNP to design locus-specific primers for amplification (i.e., type-able for genotyping). These 24-SNPs were also chosen as they were unlinked and independently segregating from each other as determined by linkage disequilibrium analysis. These 24-SNPs were verified to detect genetic diversity and population structure of 22 and 16 clinical isolates from Senegal and Thailand, respectively (Daniels et al., 2008).

#### 2.2.2 96-SNP barcode

We also examined each parasite isolate genome for their genotypes at the 96 SNPs in a genome-wide panel using the Illumina GoldenGate platform as described (Nkhoma et al., 2013). These SNPs were gleaned from PlasmoDB version 6.2 (www.plasmodb.org) and were chosen if they were highly polymorphic for parasites from the Thai-Burma border, assayable, not in genes encoding surface proteins (e.g., *var*, *rifin*, *surfin*, *stevor*), transporters or telomeric genes that may be under strong selection, were distributed across all 14 chromosomes and were found to have MAFs between 0.10 and 0.50. No formal linkage or neutrality analysis was reported in regard to the generation of the SNP barcode. The 96-SNP panel was used to analyse genetic diversity and population structure of asymptomatic and clinical isolates from pregnant women and children younger than 5 years old at the Thai-Burma border (*N* = 1,731) from 2001 to 2010 (Nkhoma et al., 2013).

### 2.3 Genotype extraction from the Pf6 database

Published positions of the 24- and 96-SNP barcodes (Daniels et al., 2008; Nkhoma et al., 2013) were based on versions 5.0 and 6.2 of the *P. falciparum* 3D7 genome on PlasmoDB (Bahl et al., 2003), respectively (Supplementary Table S1). Variants in the Pf6 database were called through read mapping to the *P. falciparum* 3D7 v3 reference genome (see Methods in (MalariaGEN et al., 2021)). Using blastn (Altschul et al., 1990), we aligned sequences containing the SNP loci of interest to the Pf3D7 v3 reference genome to obtain their corresponding positions in the Pf6 dataset. Genotypes with read depths of five or greater were retained (read depth, DP ≥ 5). In addition, alleles were only included if supported by at least two reads (allelic depth, AD ≥ 2) or 5% of reads for genotypes with higher read depths (DP > 50) (Hamilton et al., 2019). Alleles for a locus were excluded if they were single nucleotide insertions or deletions (indels), as they are strictly not defined as SNPs (Khlestkina and Salina, 2006). This excluded 0.0014% (*N* = 1) and 0.0036% (*N* = 10) of alleles in the filtered 24- and 96-SNP datasets, respectively.

### 2.4 Addressing multiple *P. falciparum* infections

#### 2.4.1 Defining monoclonal and multiclonal *P. falciparum* infections

**FIGURE 2:**
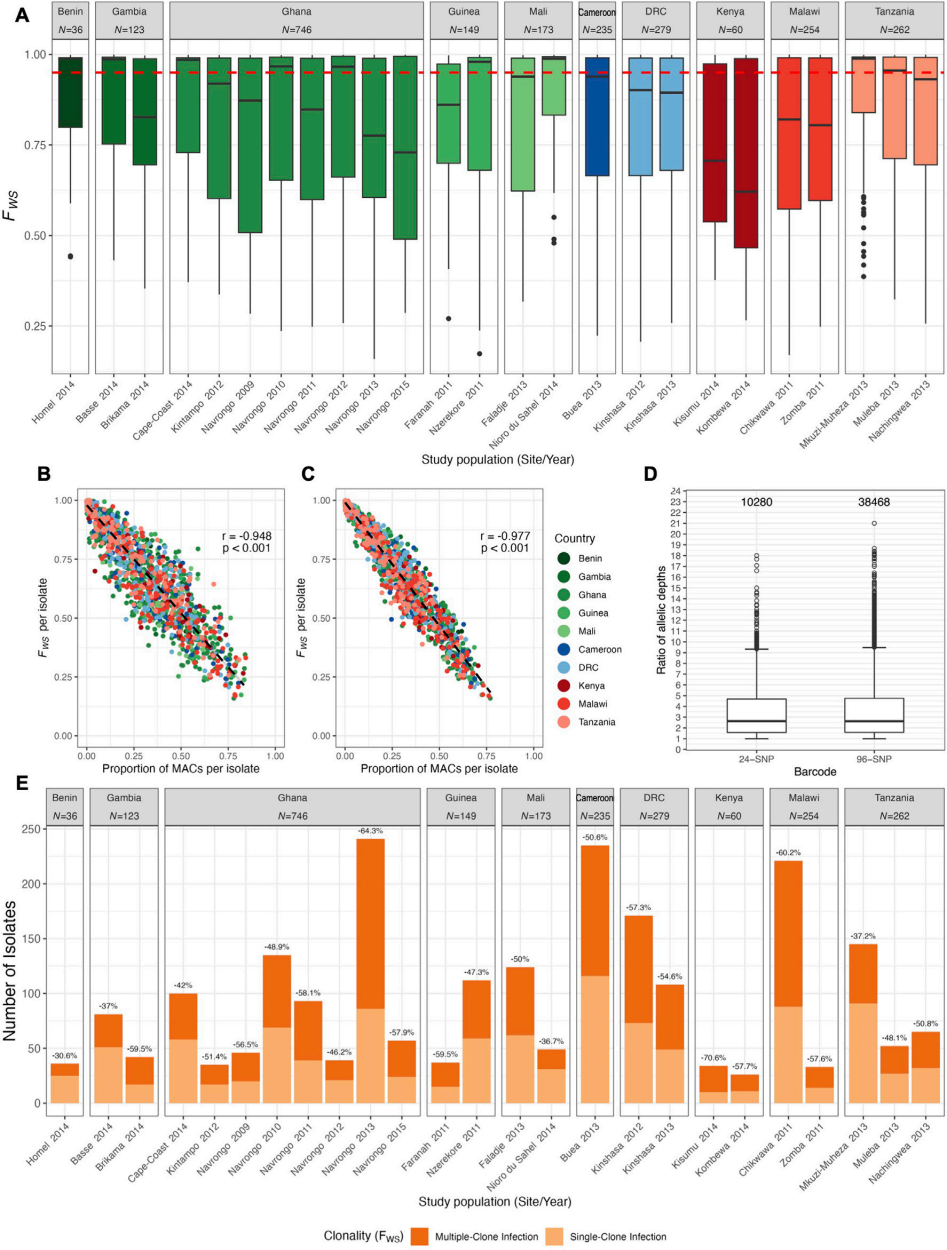
Clonality of infections in African study populations with moderate-to-high malaria transmission. **(A)** Within-host diversity using within-host inbreeding index (*F*_*WS*_). The dotted red line indicates the *F*_*WS*_ ≥ 0.95 threshold below which isolates were considered to have diverse multiclonal infections. The country for the study population is indicated for reference on the top with total number (*N*) of isolates represented for that country. **(B, C)** Correlation between *F*_*WS*_ and the proportion of mixed-allele calls (MACs) per isolate for the **(B)** 24 SNP barcode and **(C)** 96 SNP barcode. Each dot represents one isolate per study population (location by year). For the both barcodes, the *F*_*WS*_ and MACs per isolate were significantly negatively correlated (Pearson’s correlation coefficient (r) and *p*-value are shown). **(D)** Positively-skewed distributions of allelic depth ratios (AD ratio) from exploring the potential use of the “dominant allele” method. AD ratios close to one indicate approximately similar read coverage for both alleles whereas large AD ratio values represent a substantial difference in read coverage for two alleles. Numbers above the box plots represent the number of genotypes with MACs considered in these calculations. Horizontal central solid line represents the median, the box represents the interquartile range (IQR) from the 25th to 75th percentiles, the whiskers indicate the most extreme data point, which is no more than 1.5 times the interquartile range from the box, and the dots show the outliers. **(E)** Data loss in all study populations from using the “conservative” approach of excluding multiclonal infections. Total number of isolates per study population with monoclonal and multiclonal infections are shown as light and dark orange bars, respectively. Data is separated by study population (study location by year) and values above each bar indicate the percent of data lost when removing multiclonal infections (*F*_*WS*_ < 0.95) from the analysed datasets.

To determine the clonality of infections, we obtained data on the within-host inbreeding index (*F*_*WS*_) for each isolate from the Pf6 dataset (MalariaGEN et al., 2021). This metric estimates the allele frequency of parasites within an individual isolate (*H*_*W*_) relative to the allele frequency within the total parasite population (*H*_*S*_) using the read count for each locus in the Pf6 dataset. *F*_*WS*_ is presented as a proportion that ranges from 0 to 1, where *F*_*WS*_ values closer to 1 indicate high inbreeding rates (less genetically diverse) and lower *F*_*WS*_ values indicate low inbreeding rates (more diverse/mixed genotypes) in the parasite population. An infection is said to predominantly contain a single genotype when *F*_*WS*_ ≥ 0.95 (Manske et al., 2012; Mobegi et al., 2014; Duffy et al., 2018; Amambua-Ngwa et al., 2019; Amegashie et al., 2020). Based on this, *N* = 1,105 isolates were found to predominantly have a monoclonal infection (Figure 2A). To maintain study population sizes ≥25, nine study populations with <25 isolates were removed from analysis, resulting in *N* = 956 isolates (Supplementary Figure S1).

#### 2.4.2 Mixed-allele calls (MACs)

To determine whether multiclonal infections could be used for downstream population genetics analyses, we needed to ensure constructed multilocus haplotypes did not include more than 5% of the barcode with mixed-allele calls (MACs, reported as “N” in other studies e.g. (Daniels et al., 2008)). Including haplotypes with many MACs would consequently introduce a high degree of uncertainty into each haplotype and affect subsequent results. Further, in studies where whole-genome sequence data is not available, the clonality of isolates is determined by the percentage of MACs for an isolate. Isolates in which more than one allele was observed for greater than or equal to 5% of loci are conventionally termed as multiclonal infections, and monoclonal infections are those with less than 5%, e.g., (Daniels et al., 2008; Rice et al., 2016). We therefore kept a tally of the number of MACs per locus to understand the genetic complexity per locus and if it was evenly distributed. The Pearson’s correlation coefficient was calculated using the function “cor.test” in the R package “stats” v. 3.6.2, to test the association between MACs and *F*_*WS*_.

#### 2.4.3 Investigating two approaches to handling multiclonal infections

We tested two common methods of accounting for multiclonal infections in SNP or whole-genome data analysis. The first approach (i.e., “dominant allele” method) attempts to include both mono- and multiclonal infections (*N* = 2,317) in analyses by constructing the “dominant” haplotype for each isolate that has a MAC. This artificially generates a monoclonal infection for all genotypes. A “dominant” allele was defined as an allele call with the highest number of supporting reads (i.e., higher AD) per SNP locus using the ratio of AD (dividing the larger AD by the smaller AD). For loci where both alleles were supported equally (i.e., AD ratios = 1), an allele was selected at random to complete the construction of haplotypes without MACs (Manske et al., 2012). Higher AD ratio values (i.e., AD ratios >1) indicated that one allele had more supporting reads than the other.

The second more “conservative” method removes all multiclonal infections, as defined by *F*_*WS*_, retaining only monoclonal infection data for subsequent analysis (*N* = 1,105). The percentage of data loss for the latter method was calculated as the number of multiclonal infections divided by the total number of infections per study population.

### 2.5 Using the performance criteria to analyse SNP barcodes

Performance of the 24- and 96-SNP molecular barcodes were analysed to estimate genetic diversity and population structure as described below.

#### 2.5.1 Minor allele frequency (MAF) calculation

MAFs are central to analyses using SNP data and is therefore important to accurately estimate. Subsequent to our investigation of methods for handling multiclonal infections that found the conservative approach (Anderson et al., 2005; Taylor et al., 2017; Amegashie et al., 2020; Han et al., 2022) as the more stringent and reliable method, MAFs in downstream analyses were estimated using only monoclonal infections. A custom R script was used to calculate the MAFs according to the genotype data that was input (available on GitHub at: https://github.com/UniMelb-Day-Lab/SNP_MinorAlleleFreq). In short, MAFs for each locus were calculated by removing MACs from the numerator and denominator to reduce bias. This custom script generates a table describing in each row a locus with the number of isolates with data, the number of MACs, the major and minor alleles, and the minor allele frequency calculated. Because samples were haploid, Hardy-Weinberg Equilibrium was not applicable in this study.

#### 2.5.2 Spatial analysis of MAFs

A MAF <0.10 indicated that a locus was not representative and that alleles were moving towards fixation in the population, while a MAF ≥0.10 indicated that the locus can discriminate between isolates in the population. As MAFs impact the inference of population structure (Anderson et al., 2005), MAFs were analysed by region, country and study population (study location per year). Four loci were removed from the 24-SNP panel and 21 loci were removed from the 96-SNP panel as they were not strictly biallelic and/or had MAFs <0.10, resulting in 20-SNP and 75-SNP barcodes analysed downstream for informative population genetics analyses (Supplementary Figure S1).

#### 2.5.3 Testing multilocus association within SNP barcodes

The standardised index of association ( ${\bar{r}}_{d}$ ) was used to estimate the extent of multilocus linkage disequilibrium (LD) i.e., the non-random association of alleles (Agapow and Burt, 2001), across the 20- and 75-SNP barcodes. Pairwise ${\bar{r}}_{d}$ was calculated to determine whether any patterns of LD were due to any pairs of SNP loci, or if there were any significantly associated pairs of loci masked by an overall LD. If the SNP loci were putatively neutral, then multilocus LD would provide evidence of past and/or current selection on the local parasite population (Ruybal-Pesántez et al., 2017a). The ${\bar{r}}_{d}$ and pairwise ${\bar{r}}_{d}$ among loci were estimated using a Monte Carlo simulation method of 999 samplings, where alleles were reshuffled at random among haplotypes, using the R package poppr v. 2.7.1 (Kamvar et al., 2014). To calculate ${\bar{r}}_{d}$ and pairwise ${\bar{r}}_{d}$ , only isolates with complete infection haplotypes (i.e., no missing data) were used so that the permutation analysis shuffled the alleles per haplotype without bias.

#### 2.5.4 Genetic diversity

In order to calculate genetic diversity estimates of the number of multilocus haplotypes (*h*), expected heterozygosity (*H*_*e*_) and population genetics analyses, MACs were replaced with no value (“NA”), hereinafter known as the “cleaned monoclonal infections” dataset (Supplementary Figure S1). The mean values of *h* and *H*_*e*_ were calculated for both barcodes across each region, country and study population using the “cleaned monoclonal infection” haplotypes (where MACs were removed) via R package “poppr” v. 2.7.1 (Kamvar et al., 2014).

#### 2.5.5 Allelic differentiation by locus and over spatial scales

Pairwise population genetic distances across each population scale were determined by Weir and Cockerham’s *F*_*ST*_ using the R package “hierfstat” v. 0.5-10 (Winter 2012). *F*_*ST*_ is a measure of the extent an allele is fixed between populations (Jost et al., 2018), and was calculated as the proportion of allelic variance between loci for the 20- and 75-SNP genotypes. *F*_*ST*_ values range from 0 to 1, where values close to 1 indicated that populations were fixed for different alleles, while values close to 0 denote that allele frequencies were identical in both populations. Pairwise *F*_*ST*_ was used to calculate estimates of allele differentiation between pairs of regional, country and study population levels. Only cleaned multilocus haplotypes (i.e., with no missing data) were used to calculate *F*_*ST*_ and pairwise *F*_*ST*_, referred to as the “complete monoclonal infections” dataset (Supplementary Figure S1), which resulted in 653 and 690 isolates for the 20- and 75-SNP barcodes respectively. A Mantel test was calculated using the R package “vegan” v. 1.3.3 (Oksanen et al., 2020) with 999 iterations to evaluate the relationship between geographic distance (latitude and longitude, Table 1) and genetic divergence (pairwise *F*_*ST*_). Given that there were only three regions to generate a matrix, comparisons between regions could not be performed.

#### 2.5.6 Population structure analysis

Population differentiation between study populations, countries, and regions was evaluated by discriminant analysis of principal components (DAPC) using the “complete monoclonal infections” dataset of the 20- and 75-SNP barcodes (Supplementary Figure S1). DAPC is a multivariate method that aims to summarise genetic differentiation between groups and was calculated using the R package “adegenet” v. 2.1.5 (Jombart, 2008). The DAPC can detect population structure below a threshold detectable by *F*_*ST*_, providing an estimate of how much data was required to find population structure given genetic differentiation in the population (Patterson et al., 2006). Pairwise distance matrices (i.e., PCA) were first built from evaluating the proportion of SNPs that had different alleles for two isolates. The outputs were a series of uncorrelated eigenvectors (principal components) that determined the directionality of space in the PCA plot, and eigenvalues that determined the magnitude or variation of genetic diversity along the axis. Eigenvalues greater than one accounted for more variance than one of the original variables in the data. Discriminant analyses of these matrices identified the contribution of alleles to possible clusters that may have been driving genetic differentiation between populations. Ellipses were drawn that contained 95% of the genotypes per population. The term “discriminant function” (DF) was used to explain the principal components input to calculate the DAPC. Plots of DF eigenvalues and the contribution of each allele to explain population structuring were generated using “adegenet” for each corresponding DAPC (Jombart, 2008).

#### 2.5.7 Genetic similarity

To identify finer-scale levels of structure without geospatial location data for each individual isolate, we calculated the pairwise allele sharing (*P*_*AS*_) score for isolates within each study population for each barcode using the “complete monoclonal infections” haplotypes with no missing data. *P*_*AS*_ is an identity-by-state (IBS) measure of genetic similarity that can be used across relatively few loci and was calculated as the number of alleles shared between two multilocus haplotypes (*N*_*AB*_) divided by the number of SNP loci (*N*_*L*_) (*P*_*AS*_ = *N*_*AB*_/*N*_*L*_) (Ruybal-Pesántez et al., 2017a; Argyropoulos et al., 2021). The *P*_*AS*_ score characterised variation in multilocus haplotypes from clones (*P*_*AS*_ = 1.0) to genetically dissimilar (*P*_*AS*_ ≤ 0.25) (Argyropoulos et al., 2021). Larger-scale genomic measures like identity-by-descent (IBD) are performed for larger genome sequences (a minimum of 200 biallelic loci) to infer similarity or “relatedness” over a range of DNA segments (Henden et al., 2018; Schaffner et al., 2018; Taylor et al., 2019) and therefore were unable to be pursued.

#### 2.5.8 Temporal analysis of genetic diversity and similarity

Study locations with isolate data in more than one time point were used to investigate whether the SNP loci in each panel were able to be used longitudinally. Temporal data using the “cleaned monoclonal infections” dataset were available for Navrongo, Ghana (2010, 2011, and 2013) and Kinshasa, DRC (2012 and 2013) (Supplementary Figure S1). MAFs and *H*_*e*_ were compared within each study location over time using the cleaned monoclonal infections data. The function “Hs.test” in “adegenet” was used to test the difference in *H*_*e*_ between two time points (x and y) using the equation *H*_*e*_(x) - *H*_*e*_(y) using 999 Monte-Carlo test simulations (Jombart, 2008). Subsequent analysis of variation of loci on chromosome 7 of the 20-SNP barcode led to a closer investigation with its association to a known gene under selection, *Plasmodium falciparum chloroquine resistance transporter* (*pfcrt*), which may be in close proximity to these SNP loci. We obtained data on the drug resistance classification (sensitive/resistant/undetermined) and marker genotypes for each isolate from the Pf6 dataset (MalariaGEN et al., 2021). Resistance against chloroquine (CQ) and other 4-aminoquinolines, including the artemisinin drug combination amodiaquine (AQ), is primarily governed by the K76T mutation in *pfcrt* on chromosome 7. A chi-squared test (χ^2^) was used for univariate analyses of discrete variables to compare proportions. *P*_*AS*_ were compared between study locations over time using the “complete monoclonal infections” data. As such, isolates from Navrongo 2011 were removed as there were <25 complete infection haplotypes for analysis*.* A non-parametric Wilcoxon rank-sum test was used to compare the *P*_*AS*_ between two time points in Base R v. 3.5.0 (R Core Team, 2018).

### 2.6 Statistical tests

All statistical analysis were carried out in R (R Core Team, 2018) implemented in RStudio v. 1.1.383 (RStudio Team, 2015) with Base R and the R package “tidyverse” v. 1.3.1 (Wickham et al., 2019) for data curation and visualisation. A test was deemed statistically significant if the *p*-value was <0.05.

## 3 Results

### 3.1 Description of the Pf6 database study populations and epidemiology

The availability of SNP genotypes in the Pf6 database allowed us to test the performance of the 24- and 96-SNP barcodes to examine population diversity and structure. There were 2,922 isolates sampled in Africa that met the selection criteria (see Methods). Of these, haplotypes were generated for 2,317 (79%) isolates from 25 study populations (study location by year) across 10 moderate-to-high transmission countries in Africa. Study population sample sizes varied from 26 isolates (Kombewa, Kenya, 2014) to 235 isolates (Buea, Cameroon, 2013) (Table 1). There were seven study populations across three countries in East Africa (Kenya, Malawi, and Tanzania), three study populations across two countries in Central Africa (Cameroon and DRC), and the remaining 15 study populations across five countries in West Africa (Benin, The Gambia, Ghana, Guinea, and Mali) (Table 1; Figure 1). The number of isolates, MACs, major and minor alleles, and minor allele frequencies (MAFs) were generated per locus for each study population (Supplementary Tables S2, 3 for the 24- and 96-SNP barcodes, respectively). Malaria transmission in these study populations was predominantly seasonal and year-round (perennial), with few populations exhibiting double peak (two higher-transmission seasons) and unstable (large variation year-to-year) transmission (Table 1). All isolates in these studies were obtained from clinical malaria cases across all ages, from newborns to above 65 years old, with only one study collecting additional data from individuals across all ages with asymptomatic malaria infections (Table 1).

### 3.2 Majority of overall infections in African study populations were multiclonal

We investigated the clonality of infections using the within-host inbreeding index, *F*_*WS*_, where values ≥0.95 indicated that infections predominantly contained a single genome. We showed that 52.3% of overall infections were found to be multiclonal and that these multiclonal infections dominated in most study populations (Figure 2A). Similarly, more than half of infections in the 24-SNP barcode (53.0%) and the 96-SNP barcode (56.4%) had more than 5% mixed-allele calls (MACs) in a haplotype (Supplementary Table S4), which is the threshold typically used to determine clonality of infections (see Methods). Comparisons of *F*_*WS*_ values to proportions of MACs for each same infection revealed a significant negative correlation between the two metrics for both barcodes (24-SNP: *r* = -0.948 [95% CI: −0.952, −0.944], *p* < 0.001; 96-SNP: *r* = -0.977 [95% CI: −0.979, −0.975], *p* < 0.001), demonstrating that proportions of MACs in an isolate’s haplotype is a reliable predictor of within-host diversity for an isolate for cases where *F*_*WS*_ is unavailable (Figures 2B,C).

Given that such large proportions of infections in all study populations were reported as multiclonal, we further explored two prevailing approaches that have been used in the literature to either include or exclude multiclonal infections in downstream analyses (see Methods for detailed descriptions of both approaches). For the “dominant allele” method, distributions of AD ratios were both positively skewed for both barcodes (Figure 2D). The median of AD ratios for genotypes with MACs of the 24-SNP and 96-SNP barcode was 2.63 (IQR: 1.58-4.68) and 2.62 (IQR: 1.59-4.75), respectively, indicating that most MACs were due to alleles that were found in approximately similar proportions. Given this result, the use of this “dominant allele” method potentially introduces uncertainty in downstream calculations of MAF as the assignments of most alleles would be at random or possibly confounded by systematic biases in read coverage. This poses the risk of reconstructing inaccurate haplotypes for the majority of infections.

Consequently, we chose to perform all subsequent analyses using the “conservative” approach of excluding isolates with multiclonal infections (*F*_*WS*_ < 0.95). While this approach ensured a higher confidence in the constructed haplotypes, the result was a reduction in the total number of isolates from 2,317 to 1,105 (Supplementary Figure S1). When inspected by study populations, the exclusion of multiclonal infections resulted in data loss for every study population analysed (Figure 2E). The smallest reduction in the number of isolates was observed for Homel 2014, Benin (30.6% of infections) whereas the largest reduction in the number of isolates was reported for Navrongo 2013, Ghana (64.3% of infections).

### 3.3 Criteria I and II: low minor allele frequencies (MAFs) and non-biallelic nature of multiple SNP loci resulted in reduced barcode sizes and lower expected heterozygosity

The monoallelic, triallelic, and multiallelic loci observed in >70% of the study populations were removed from downstream analysis, resulting in 20-SNP and 81-SNP barcodes (Supplementary Table S5). See Supplementary Results section 1.2.2 for a detailed description of the observed polymorphisms in the two molecular barcodes. Using the “cleaned monoclonal infections” dataset, six loci in the 81-SNP barcode had MAFs below 0.10 (Supplementary Table S6), indicating that these loci would not be informative to differentiate isolates from each other in the population. These loci were removed from downstream analyses, resulting in a 20-SNP and 75-SNP barcode, respectively. Table 2 shows MAFs by region, country, and study population. The median MAF across all loci was 0.352 (IQR: 0.254-0.422) and 0.333 (IQR: 0.234-0.419) for the 20- and 75-SNP panels, respectively, across all 25 study populations, and was similar across all regions, countries, and study populations for both barcodes (Table 2).

**TABLE 2:** Patterns of *P. falciparum* genetic diversity of monoclonal infections in African study populations in Pf6 database for the 20- and 75-SNP barcodes.

	*N*	*h*		*H*_*e*_		MAFs	
Population		20-SNP	75-SNP	20-SNP	75-SNP	20-SNP	75-SNP
***West Africa***				**0.425**	**0.429**		
**Benin**				**0.432**	**0.421**	**0.320 [0.270–0.410]**	**0.320 [0.208–0.400]**
Homel 2014	25	25	25	0.432	0.421	0.320 [0.270–0.410]	0.320 [0.208–0.400]
**The Gambia**				**0.411**	**0.433**	**0.326 [0.186–0.452]**	**0.333 [0.255–0.431]**
Basse 2014	51	45	45	0.411	0.433	0.326 [0.186–0.452]	0.333 [0.255–0.431]
**Ghana**				**0.422**	**0.427**	**0.345 [0.258–0.423]**	**0.337 [0.240–0.419]**
Cape-Coast 2014	58	57	57	0.422	0.417	0.362 [0.272–0.448]	0.293 [0.198–0.400]
Navrongo 2010	69	69	69	0.426	0.428	0.350 [0.247–0.407]	0.348 [0.261–0.398]
Navrongo 2011	39	39	39	0.408	0.439	0.315 [0.250–0.433]	0.359 [0.266–0.436]
Navrongo 2013	86	84	84	0.421	0.422	0.345 [0.262–0.384]	0.349 [0.238–0.424]
**Guinea**				**0.407**	**0.432**	**0.327 [0.239–0.458]**	**0.373 [0.239–0.436]**
Nzerekore 2011	59	58	59	0.407	0.432	0.327 [0.239–0.458]	0.373 [0.239–0.436]
**Mali**				**0.436**	**0.429**	**0.381 [0.226–0.421]**	**0.355 [0.230–0.419]**
Faladje 2013	62	62	62	0.435	0.427	0.377 [0.280–0.407]	0.355 [0.232–0.419]
Nioro du Sahel 2014	31	31	31	0.442	0.436	0.392 [0.226–0.452]	0.355 [0.226–0.419]
***Central Africa***				**0.439**	**0.431**		
**Cameroon**				**0.446**	**0.425**	**0.353 [0.287–0.429]**	**0.336 [0.234–0.408]**
Buea	116	113	112	0.446	0.425	0.353 [0.287–0.429]	0.336 [0.234–0.408]
**Democratic Republic of Congo (DRC)**				**0.430**	**0.430**	**0.345 [0.261–0.417]**	**0.336 [0.247–0.429]**
Kinshasa 2012	73	72	72	0.436	0.430	0.388 [0.268–0.431]	0.356 [0.243–0.434]
Kinshasa 2013	49	47	48	0.420	0.429	0.310 [0.261–0.366]	0.327 [0.265–0.418]
***East Africa***				**0.436**	**0.420**		
**Malawi**				**0.421**	**0.416**	**0.358 [0.244–0.409]**	**0.310 [0.228–0.409]**
Chikwawa 2011	88	86	86	0.421	0.416	0.358 [0.244–0.409]	0.310 [0.228–0.409]
**Tanzania**				**0.441**	**0.419**	**0.354 [0.259–0.411]**	**0.312 [0.198–0.406]**
Mkuzi-Muheza 2013	91	82	83	0.436	0.415	0.380 [0.201–0.426]	0.333 [0.222–0.418]
Muleba 2013	27	26	26	0.436	0.415	0.321 [0.259–0.416]	0.333 [0.222–0.434]
Nachingwea 2013	32	32	32	0.439	0.429	0.344 [0.281–0.406]	0.312 [0.188–0.375]
**Total**	**956**	**925**	**929**	**0.433**	**0.432**	**0.352 [0.254–0.422]**	**0.333 [0.234–0.419]**

*h* = multilocus haplotypes; *H*_*e*_ = mean expected heterozygosity; *MAF* = minor allele frequency.

H_e_ and MAF are provided for each study population, country and region; MAF are presented as medians with interquartile ranges (IQRs). Bold values signify the rows that correspond to regions and countries.

There were 96.8% and 97.2% unique multilocus haplotypes (*h*) observed in the 20-SNP and 75-SNP molecular barcodes, respectively, using the “cleaned monoclonal infections” dataset for all locations (Table 2; Supplementary Figure S2). Of the haplotypes that were repeated, they were only found in two or three isolates for both barcodes in Basse 2014, The Gambia and Mkuzi-Muheza 2013, Tanzania (Supplementary Figure S2). Despite finding many unique haplotypes, the mean expected heterozygosity (*H*_*e*_) was low when using both barcodes (20-SNP: *H*_*e*_ = 0.433; 75-SNP: *H*_*e*_ = 0.432) (Table 2) and did not vary between regions (Kruskal–Wallis: *p* = 0.368; *p* = 0.368), countries (Kruskal–Wallis: *p* = 0.444; *p* = 0.444) nor study populations (Kruskal–Wallis: *p* = 0.451; *p* = 0.451). This is best explained by the low minor allele frequencies for individual loci per barcode across the continent.

### 3.4 Criteria III: overall, loci in the 20- and 75-SNP barcodes were found to be independently segregating from each other

The standardised index of association was used to assess multilocus linkage disequilibrium (LD), or non-random associations among SNP loci, using “complete monoclonal infection” haplotypes with no missing data. Overall, there was no evidence of linkage disequilibrium for both SNP barcodes ( ${\bar{r}}_{d}$ : *p* < 0.05, Supplementary Table S7). However, at the regional-, country- and study population scale, there was significant LD when using the 20-SNP barcode in Basse 2014 (The Gambia), Cape-Coast 2014 and Navrongo 2013 (Ghana), and when using the 75-SNP barcode in Basse 2014 (The Gambia), Navrongo 2010 and 2013 (Ghana), Kinshasa 2013 (DRC) and Mkuzi-Muheza 2013 (Tanzania) (Supplementary Table S7). For the 20-SNP barcode, significant pairwise ${\bar{r}}_{d}$ values (*p* < 0.05) were found in 63 pairs of loci across all populations; the most common pairs were Pf3D7_02_v3_842805 vs. Pf3D7_10_v3_1402510, and Pf3D7_07_v3_628392 vs. P3D7_10_v3_82375 that were observed in only 3/63 pairs (4.76%) each (Supplementary Table S8). For the 75-SNP barcode, significant pairwise ${\bar{r}}_{d}$ (*p* < 0.05) was found in 1,179 pairs of loci across all populations, with the most common pair, Pf3D7_06_v3_1184506 vs. Pf3D7_06_v3_1206498, found in only 12/1,179 pairs (1.02%), indicating weak evidence of physical linkage of two markers on chromosome 6 (Supplementary Table S9). Overall, there was no evidence of prevalent LD when using the 20- and 75-SNP barcodes in these populations.

### 3.5 Criteria IV: genetic differentiation over geographic space found to be consistent with isolation-by-distance despite high genetic similarity (*P*_*AS*_)

**FIGURE 3:**
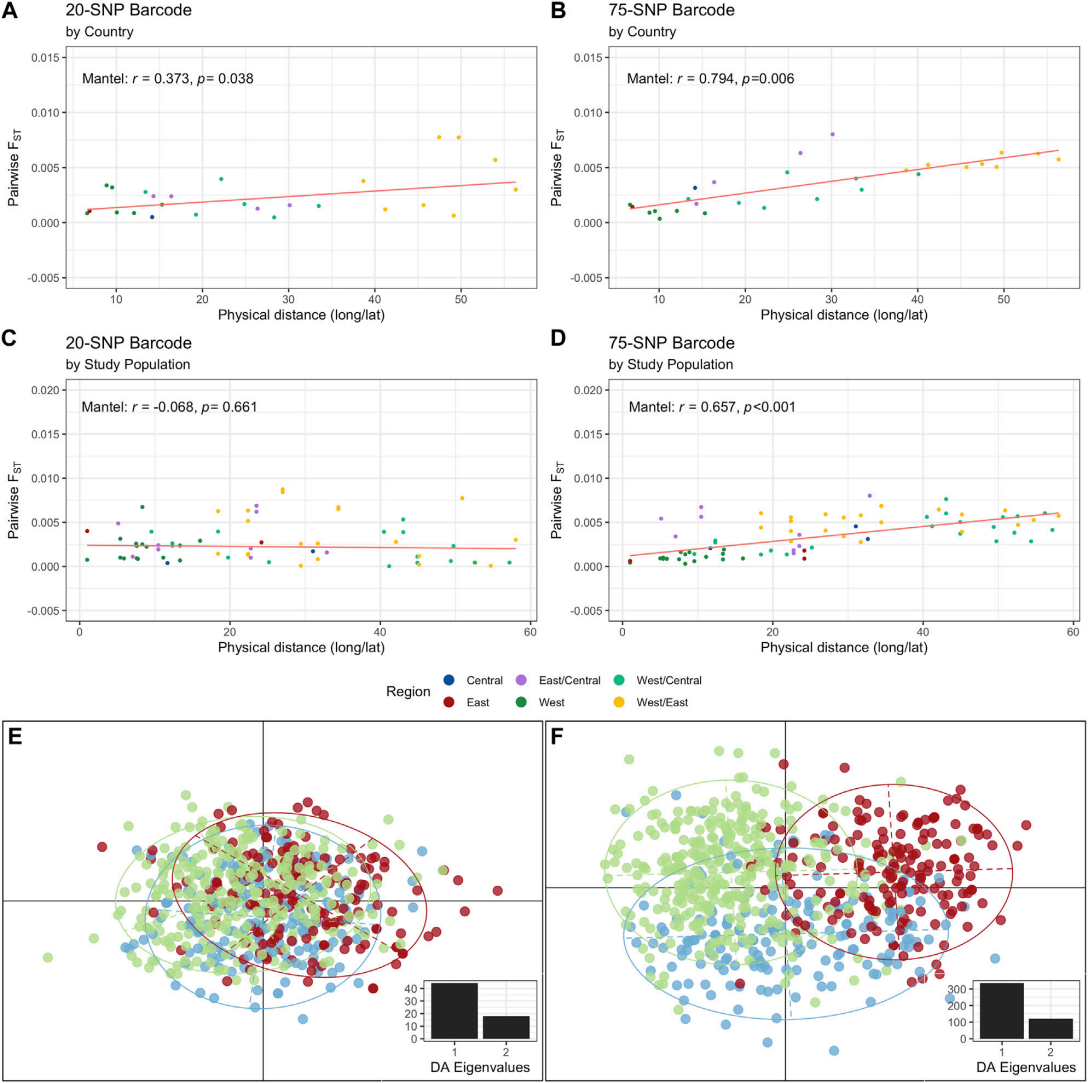
Pairwise allelic differentiation for monoclonal infections using the 20-SNP and 75-SNP barcodes. Pairwise *F*_*ST*_ values between West, Central and East Africa were calculated from *N* = 653 and *N* = 690 isolates for the 20- and 75-SNP barcodes, respectively. Pairwise *F*_*ST*_ was calculated as the proportion of allelic variance for the **(A, C)** 20- and **(B, D)** 75-SNP genotypes by **(A, B)** country or **(C, D)** by study population (study location per year). The Mantel’s test (r) and *p*-values are indicated for each SNP panel and population level (country and study population). Further, a discriminant analysis of principal components (DAPC) based on monoclonal infections with no missing data for the **(E)** 20-SNP and **(F)** 75-SNP barcode are shown. Two eigenvalues were used to plot the DAPC, as indicated in the bottom insert. The plot can be segregated into three regions (ellipticals): West Africa (green dots), Central Africa (blue dots), and East Africa (red dots).

We investigated the level of allelic differentiation by calculating pairwise Weir and Cockerham’s *F*_*ST*_ between regions, countries, and study populations using the complete monoclonal infections dataset (i.e., no missing data, Supplementary Figure S1). Overall, *F*_*ST*_ was low for each locus for the 20-SNP (mean *F*_*ST*_: 0.0165) and 75-SNP (mean *F*_*ST*_: 0.00339) barcodes (Supplementary Table S10) and pairwise *F*_*ST*_ values were very low across regions, countries, and study populations per SNP barcode (Supplementary Figure S3). The greatest genetic differentiation was between East and West Africa (20-SNP: *F*_*ST*_ = 0.0026, 75-SNP: *F*_*ST*_ = 0.0046) at the regional-level, between Guinea and Tanzania (20-SNP: *F*_*ST*_ = 0.0078) and Malawi and Cameroon (75-SNP: *F*_*ST*_ = 0.0080) at the country-level, and between Nzerekore 2011 (Guinea) and Mkuzi-Muheza 2013 (Tanzania) (20-SNP: *F*_*ST*_ = 0.0087) and Chikwawa 2011 (Malawi) and Buea 2013 (Cameroon) (75-SNP: *F*_*ST*_ = 0.0080) at the study population level (Supplementary Figure S3). Genetic and geographic variation were found to be positively correlated, signifying that genetic variation increased across greater geographic distance and *vice versa*, by country (20-SNP: Mantel: *r* = 0.373, *p* = 0.038, Figure 3A; 75-SNP: Mantel: *r* = 0.794, *p* = 0.006; Figure 3B) and by study population (75-SNP: Mantel: *r* = 0.657, *p* < 0.001, Figure 3D), consistent with a pattern of isolation-by-distance, except for the 20-SNP barcode at the study population level (Mantel: *r* = -0.068, *p* = 0.661, Figure 3C).

DAPC was used to explore the extent of population structure of *P. falciparum* across the African continent using “complete monoclonal infection” haplotypes. All principal components of the PCA were retained during the preliminary variable transformation which accounted for 100% of the total genetic variability. Genetic structure was captured by the first two DFs for the 20-SNP (Figure 3E, inset) and 75-SNP (Figure 3F, inset) barcodes. The first DF separates West and East Africa, and the second DF separates Central Africa from West and East Africa. The same patterns were reflected when the DAPCs were calculated with prior information for the country and study population per isolate for the 20-SNP (Supplementary Figures 4A, B) and 75-SNP (Supplementary Figures 4C, D) barcodes. We observed a sharp decrease in DFs when calculating DAPC by country and study populations for both barcodes, but ellipses were removed due to high overlap, indicating that smaller scale structure was not as easily identifiable (Supplementary Figure S4).

**FIGURE 4:**
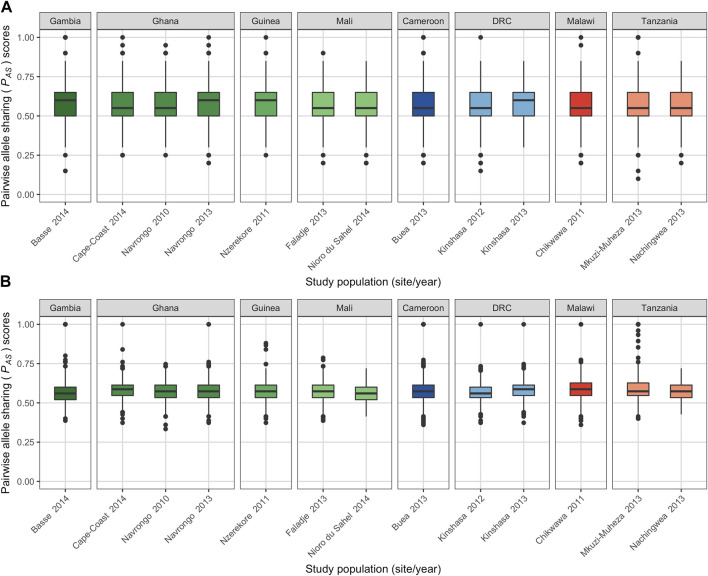
Genetic similarity within each study population using the **(A)** 20-SNP and **(B)** 75-SNP barcodes. 653 and 690 complete multilocus monoclonal haplotypes (i.e., with no missing data) for the 20- and 75-SNP barcodes were used to calculate the pairwise allele sharing (P_AS_) scores comparing isolates within each study population. There were 18,346 and 20,266 pairwise comparisons between haplotypes using the 20- and 75-SNP barcodes, respectively (see Supplementary Table S11). Colours represent populations in West Africa (green hues), Central Africa (blue hues) and East Africa (red hues). Horizontal central solid line represents the median, the box represents the interquartile range (IQR) from the 25th to 75th centiles, the whiskers indicate the most extreme data point, which is no more than 1.5 times the interquartile range from the box, and the dots show the outliers.

To understand local population structure, we calculated the genetic similarity of barcode haplotypes within the same study population using *P*_*AS*_ scores, an IBS method. To minimise bias, “complete monoclonal infection” haplotypes with no missing data were used to generate *P*_*AS*_ scores (Supplementary Figure S1). Across each study population, using both 20- and 75-SNP barcodes, we found that the majority of infection haplotypes shared more than 50% of their alleles (20-SNP: median *P*_*AS*_ = 0.550; 75-SNP: median *P*_*AS*_ = 0.573) (Figure 4; Supplementary Table S11). Using the 75-SNP barcode, we saw an absence of isolate pairs that did not share any alleles (i.e., *P*_*AS*_ = 0) and very few (8.9%) sharing up to 50% of alleles (0.2 ≤ *P*_*AS*_ < 0.5) (Supplementary Figure S12).

### 3.6 Criteria V: temporal analysis found an interchange of major and minor alleles for many loci and dynamic *P*_*AS*_ scores

**FIGURE 5:**
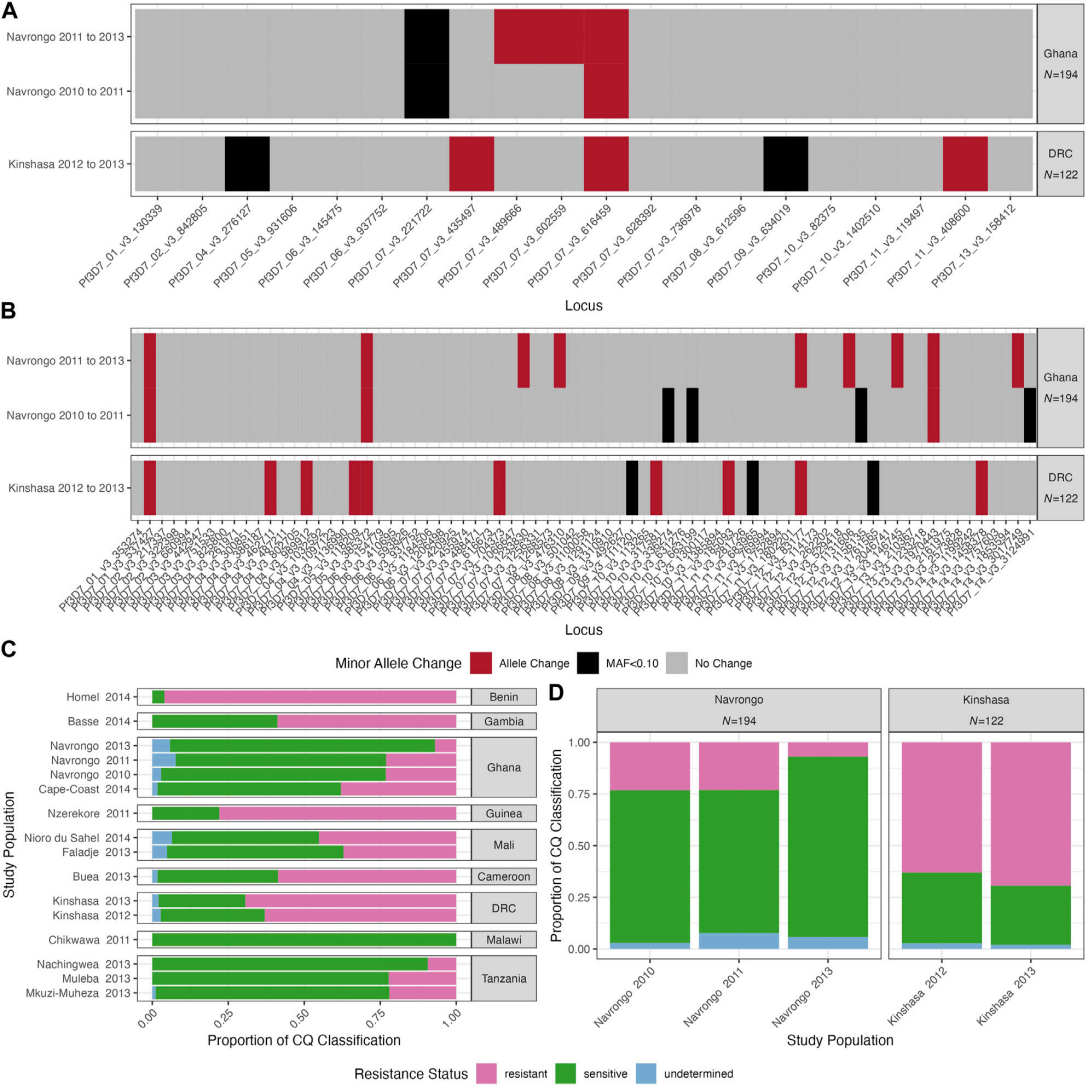
Longitudinal changes in two *P. falciparum* populations over time with monoclonal infections and chloroquine (CQ) resistance in Africa over time. Temporal study locations include Navrongo, Ghana (2010, 2011 and 2013) and Kinshasa, DRC (2012 and 2013). Changes in the minor allele using **(A)** 20-SNP and **(B)** 75-SNP barcodes are shown for Navrongo, Ghana and Kinshasa, DRC, and include interchanging base from 1 year to the next (red), changes from or to a minor allele frequency (MAF) below 0.10 approximating fixation at that locus (black), and no base or significant MAF change over time (grey). Chloroquine (CQ) drug resistance patterns are shown **(C)** within each study population and **(D)** temporal study locations. Note, colours correspond to the classification on the Pf6 database for resistance (pink), sensitive (green), and undetermined (blue). See methods for resistance classification for these drugs.

Given the likelihood of high outcrossing in these moderate-to-high transmission settings, we investigated the trends of the SNP barcodes over time. We analysed the two study locations with available temporal data: Navrongo, Ghana (2010, 2011 and 2013) and Kinshasa, DRC (2012 and 2013). Firstly, we used the “cleaned monoclonal infections” dataset to investigate whether the genetic diversity was stable over time. The mean *H*_*e*_ values were not significantly different over time (*Hs* test: *p* > 0.05, Table 3). MAFs across loci were similar over time in Navrongo and Kinshasa for both 20- and 75-SNP barcodes (Supplementary Tables S2, 3). Interestingly, for 8/20 and 23/75 SNP loci, respectively, the nucleotide base that was defined as the minor allele changed to the major allele from one year to the next in both Navrongo and Kinshasa (Figure 5). There were five SNP loci with interchanging bases on chromosome 7 for the 20-SNP barcode (Figure 5A), while the 75-SNP barcode had loci with interchanging bases spread across 10 of the 14 chromosomes (Figure 5B). Therefore, we analysed the level of drug resistance marker *pfcrt* that is also found on chromosome 7, moderated by the K76T mutation, in the two study locations over time. For *pfcrt*, 59.3% of the overall African population included in this study had the sensitive K76 allele (Figure 5C). Over time, we observed near fixation of this allele in Navrongo and Kinshasa (Figure 5D). Only the Pf3D7_07_v3_435497 ‘A’ allele was significantly related to the prevalence of CQ sensitivity in Navrongo and Kinshasa (χ^2^: *p* < 0.001, Supplementary Table S13) and should be reconsidered for population genetic analyses using neutral theory.

**TABLE 3:** Temporal changes in genetic diversity (expected heterozygosity, *H*_*e*_) and genetic similarity (pairwise allele sharing scores, *P*_*AS*_) in Navrongo, Ghana (2010, 2011, and 2013) and Kinshasa, Democratic Republic of Congo (DRC) (2012 and 2013) for the 20- and 75-SNP barcodes.

		*H*_e_^*^		*P*_*AS*_ ‡	
Study populations over time		20-SNP	75-SNP	20-SNP	75-SNP
Navrongo (Ghana) 2010 and 2013		0.656	0.500	0.032	0.078
	2010 and 2011	0.125	0.494		
	2011 and 2013	0.204	0.465		
Kinshasa (DRC) 2012 and 2013		0.135	0.631	0.013	<0.001

*H*_*e*_ = expected heterozygosity, *P*_*AS*_ = pairwise allele sharing, *N* = number of isolates.

*Data are presented as the *p*-value calculated by “Hs.test” function.

‡ Data are presented as the *p*-value calculated by Wilcoxon test.

*H*_*e*_ was calculated using all multilocus haplotypes for both 20- and 75-SNP, barcodes: Navrongo *N* = 194, Kinshasa *N* = 122.

*P*_*AS*_ was calculated using complete multilocus haplotypes (no missing data): 20-SNP: Navrongo *N* = 108, Kinshasa *N* = 86; 75-SNP: Navrongo *N* = 120, Kinshasa *N* = 81. Navrongo 2011 was removed as there were ≤25 complete infection haplotypes for analysis.

Moreover, to understand whether isolates were genetically similar over time, we calculated the *P*_*AS*_ scores between study locations over time using the “complete monoclonal infections” data. *P*_*AS*_ scores were significantly different in Kinshasa over time (Wilcoxon: 20-SNP: *p* = 0.031 and 75-SNP: *p* < 0.001) and in Navrongo from 2010 to 2013 for the 20-SNP barcode (Wilcoxon: *p* = 0.032) but not the 75-SNP barcode (Wilcoxon: *p* = 0.078) (Table 3).

## 4 Discussion

Here we present the first study to critically evaluate the use of two SNP barcodes in moderate-to-high transmission countries in Africa with a high proportion of multiclonal *P. falciparum* infections. Both 24- and 96-SNP barcodes could recapitulate a signal of large-scale genetic differentiation by geographic distance as shown by Mantel Test and DAPC, consistent with minimal divergence of loci with high gene flow across the African continent (Mobegi et al., 2012; Mobegi et al., 2014; Duffy et al., 2017; MalariaGEN et al., 2021). But finer-scale estimates of genetic diversity (*H*_*e*_) and similarity (*P*_*AS*_) were not reflective of highly outcrossing populations, likely because these small molecular barcodes were not strictly biallelic and had similar and low minor allele frequencies (Table 4). Although multilocus SNP haplotypes were found to be largely unique, they only differed at one or two loci. This paucity of informative loci led to the erroneous conclusion that they appeared to be clonal or genetically similar, which may result in a less suitable solution to control. Additionally, analysing two study locations with temporal data showed that the allele frequencies per locus changed rapidly over short one-year periods, concordant with a large effective population size and high outcrossing rates (Anderson et al., 2000). Our results highlight two key points for SNP barcodes in moderate-to-high transmission settings in Africa, i) the high number of multiclonal infections led to approximately half of the data loss and ii) the low minor allele frequencies across SNP loci biased genetic diversity and population genetic estimates.

**TABLE 4:** Summary of SNP barcode performance in Africa to determine the genetic diversity and population structure of *P. falciparum*.

Metric of interest	Purpose	24-SNP barcode	96-SNP barcode
Clonality (mono- or multiclonal)	To perform downstream analyses on genetic diversity and population structure	Only useful for MOI = 1 (Due to high MACs >5%); >50% of isolates removed from data analysis	Only useful for MOI = 1 (Due to high MACs >5%); >50% of isolates removed from data analysis
Biallelic (polymorphism)	Common assumption and is required to capture variation	Must remove 16.7% loci: 20-SNP barcode	Must remove 25% of loci: 81-SNP barcode
Minor Allele Frequencies (MAFs) > 0.10	Required to capture variation	*No further SNPs removed*	Must remove 7.5% of loci: 75-SNP barcode
Independent segregation of loci/selectively neutral	Variation maintained irrespective of areas under genetic selection	SNPs on chromosome 7 may be in LD to *pfcrt*	Yes
Genetic diversity (*H*_*e*_)	Reflects variation in the gene pool of population	Due to low MAFs, *H*_*e*_ is also lower	Due to low MAFs, *H*_*e*_ is also lower
Spatial genetic differentiation (*F*_*ST*_)	Reflects local evolutionary history of the populations	Association at low resolution	Association with slightly better resolution
Genetic similarity (*P*_*AS*_)	Detect presence of clonal/similar parasites (clone outbreak)	Due to low *H*_*e*_, *P*_*AS*_ is higher	Due to low *H*_*e*_, *P*_*AS*_ is higher with smaller IQR
Temporal stability	Similar allele frequencies maintained over time for longitudinal studies	MAFs change over one-year	MAFs change over one-year

Abbreviations: MOI = multiplicity of infection; MACs = mixed allele calls; LD = linkage disequlibrium; MAF = minor allele frequency.

Biallelic SNP markers have proven highly informative in molecular surveillance for *P. falciparum* in low-transmission settings. But our results underline that in moderate-to-high transmission settings, where the number of multiclonal infections outweighs monoclonal infections, the use of SNP barcodes as a molecular marker for surveillance is constrained. We demonstrated that reconstructing haplotypes from assigning a dominant allele is random as alleles in a mixed infection are found at equal proportions. This led to only retaining monoclonal infections, removing half of the isolate data to perform reliable genetic diversity and population genetics analyses. This is concerning due to possible introduced bias in reducing sample size when performed in the real-world, seen with our case study in Obuasi where only approximately 15%–20% of the surveyed population had monoclonal infections. This is further exacerbated when accounting for the cost of equipment, reagents, and labour involved in the data generation. An additional cost that has not been considered is the need to survey large numbers of individuals to get enough monoclonal infections. The lack of useable data differs from many other scenarios in the literature where SNPs have been used in low-transmission regions with predominantly monoclonal infections.

While software packages such as THE REAL McCOIL (Chang et al., 2017), DEploid (Zhu et al., 2018), and DEploidIBD (Zhu et al., 2019) attempt to phase or reconstruct SNP datasets with multiclonal infections using Bayesian and/or Markov Chain Monte Carlo methods, they introduce a large degree of uncertainty and assumptions, particularly when there are three or more genotypes per infection with a high number of MACs (Labbé et al., 2023). In fact, in areas of such high transmission and endemicity, it is not uncommon for infections to contain five or more distinct *P. falciparum* clones per microlitre of blood (Chang et al., 2017; Tiedje et al., 2017; 2022; World Health Organisation, 2018). This drawback extends to larger SNP-based panels (>500 SNPs) due to the high occurrence of MACs, frequent outcrossing, and large effective population size in high-transmission settings. For example a study by Verity et al. (2020) sequenced 2,537 isolates in the Democratic Republic of Congo, Ghana, Tanzania, Uganda and Zambia using a panel of 739 geographically informative SNPs and another panel of 1,151 putatively neutral SNPs across the *P. falciparum* genome. Of these isolates, only 1,382 (54.5%) and 674 (26.6%) respectively passed the quality control and filtering steps, resulting in an enormous loss of data and expense. These issues of cost-effectiveness are of relevance to public health where only approximately $1-10 per person per annum is spent on malaria control in endemic countries in Africa (World Health Organisation, 2022).

Of the remaining monoclonal samples that were able to be analysed, our results from SNP barcodes did not reflect diversity, similarity and structure estimates as found in other studies using a higher magnitude of genome-wide SNPs (Mobegi et al., 2014; Daniels et al., 2015; Amambua-Ngwa et al., 2019; Moser et al., 2020; Verity et al., 2020; MalariaGEN et al., 2021), putatively neutral microsatellites (Anderson et al., 2000; Mobegi et al., 2012; Duffy et al., 2017; Argyropoulos et al., 2021) and antigenic markers (Ruybal-Pesántez et al., 2017b; Day et al., 2017; Rorick et al., 2018). One possible explanation for these observed discrepancies is the “ascertainment bias” phenomenon, where polymorphisms that were discovered in few samples or locations can result in a deviation from an expected allele frequency distribution (Kuhner et al., 2000; Wakeley et al., 2001; Helyar et al., 2011). While these loci were polymorphic in Senegal and Thailand (24-SNP barcode (Daniels et al., 2008)) and along the Thai-Burma border over 10 years (96-SNP barcode (Nkhoma et al., 2013)), when applied to these African populations, some loci were mono or triallelic, indicating fixation or hypermutable sites respectively, and other loci had low average MAFs than would be useful. This consequently biases estimates which rely on allele frequencies, such as expected heterozygosity, linkage disequilibrium, genetic similarity, and population structure (Wakeley et al., 2001; Nielsen and Signorovitch, 2003; Helyar et al., 2011; Speed and Balding, 2015; Taylor et al., 2019) To minimise these biases and for barcodes to potentially work across multiple populations, loci must be carefully selected by local- and large-scale geospatial sampling and whole-genome sequencing of multiple isolates (Helyar et al., 2011); if these loci were to be analysed using neutral theory, as with these barcodes discussed, then these SNP loci must also be assessed for signals of selection (e.g., using Tajima’s *D*). A study of this magnitude is currently very expensive (approximately $86 USD per isolate) (Tessema et al., 2020), laborious, and is not guaranteed to produce a SNP barcode that is temporally stable, particularly in highly recombining settings (as reviewed in (Escalante et al., 2015)), due to the profound effects of sexual recombination.

Longitudinal investigations using SNP barcodes must err on the side of caution. Given the recent changes in antimalarial drug policy and use (World Health Organisation, 2022), it is possible that selection of the K76 allele of *pfcrt* (i.e., chloroquine sensitivity) is driving variation at Pf3D7_07_v3_435497 in the 20-SNP barcode. This observation corresponds to the policy change to artemether-lumefantrine (AL) and artesunate-amodiaquine (ASAQ) in Ghana in 2007, where reports have indicated a higher use of AL (World Health Organisation, 2015) that selects for the K76 allele (Sisowath et al., 2009; Venkatesan et al., 2014); increased prevalence of K76 has also been reported in a nearby region of Bongo District, Ghana (Narh et al., 2020). In DRC, there has been low yet steady increase in ACT use from 2% in 2010 to 30% in 2017–2018 (U.S. President’s Malaria Initiative, 2020), coinciding with the slow increase in chloroquine sensitivity (*pfcrt* K76). This provides an example of how important longitudinal investigations of molecular panels are to ensure population genetics theories are being upheld. Any temporal variation in allele frequencies related to outcrossing must complicate the calculation of priors for Bayesian inference.

A key assumption when analysing SNPs in population genetics is that they are biallelic (Schlötterer, 2004), but as shown when using the whole-genome sequence data to generate our barcode haplotypes, this is not always the case. The two SNP barcodes used in our analysis, however, were designed to be genotyped using platforms that are only able to detect two previously identified bases (alleles) per locus (e.g., Taq-Man or Illumina GoldenGate). How then can we monitor genetic diversity and population structure in moderate-to-high transmission settings? The answer likely lies in the use of polymorphic markers such as putatively neutral markers that permit the inclusion of “dominant” infections (e.g., short tandem repeats (STRs) or microsatellites) (Anderson et al., 1999; Tessema et al., 2020). For example, microsatellites were able to resolve global *P. falciparum* structure with only 12 markers (Anderson et al., 2000), while 9-10 microsatellite markers were able to give realistic assessments of these measures related to both long-lasting insecticidal net (LLIN) (Kattenberg et al., 2019) and indoor residual spraying (IRS) (Argyropoulos et al., 2021) interventions, respectively, in moderate-to-high transmission settings. With respect to neutral variation, STR loci are more useful to detect recent population expansions than SNPs as they accumulate new mutations at a faster rate, are multiallelic often in excess of 10 alleles, and have more private alleles; thus they remain the most informative putatively neutral markers in population genetic studies across many organisms (Ellegren, 2004; Selkoe and Toonen, 2006; Guichoux et al., 2011), including in *P. falciparum* and *P. vivax* genomes across various geographic populations (Han et al., 2022). Microhaplotypes, regions of 100–200 bp with high genetic diversity unbroken by recombination, of SNPs and STR loci are currently proposed as a high-throughput and automated alternative to microsatellite genotyping methods that rely on capillary electrophoresis (Tessema et al., 2020). However current microhaplotype genotyping for *P. falciparum* is largely SNP-based and yet to be deployed in high-transmission settings in the field.

Alternatively, adaptive genes may present an innovative approach (Barton, 2010) consistent with the large parasite population size seen within and between human hosts in sub-Saharan Africa. Antigenic markers, which rely on size- or coding-sequence polymorphisms (e.g., *msp2*, *csp*, *ama1*, *var*), can distinguish highly diverse multiclonal infections, but cannot construct haplotypes (Snounou et al., 1999; Ruybal-Pesántez et al., 2017b; Nelson et al., 2019). A recent study (Ghansah et al., 2023) compared the use of SNPs, microsatellites and *var* DBLa typing (*“var*coding”) to evaluate genetic diversity and population structure in a high-transmission setting in Ghana and found that while microsatellites provided greater resolution than SNPs, *var*coding was superior in identifying finer-scale relatedness and population structuring.

Molecular barcodes are a practical and low-cost solution to avoid relying on whole-genome sequencing for surveillance. However here we show the application of SNP barcodes encounters challenges in sub-Saharan Africa in moderate-to-high transmission settings due to the high number of multiclonal infections, frequent outcrossing, and large effective population size of *P. falciparum* as well as spatial and temporal variation. Alternative markers such as STRs and microhaplotypes are possible solutions to study *P. falciparum* population structure using neutral theory.

## Data Availability

The datasets presented in this study can be found in online respositories. The names of the repository/repositories and accession number(s) can be found at: https://datadryad.org/stash/dataset/doi:10.5061/dryad.zw3r228bc, and https://www.malariagen.net/resource/26.
