# Supplementary material for: Performance of SNP barcodes to determine genetic diversity and population structure of Plasmodium falciparum in Africa
Source: Front Genet. 2023 Jun 1;14:1071896. doi: 10.3389/fgene.2023.1071896 (PMC10267394; doi:10.3389/fgene.2023.1071896)
Supplement: Supplementary file 3 [file DataSheet5.pdf]

1 Supplementary Material

1.1 Supplementary Methods

1.1.1 Obuasi Ethics Approval

The study was reviewed/approved by the ethics committees at the Noguchi Memorial Institute for Medical Research Institutional Review Board, with certified protocol number CPN 11/04-05.

1.1.2 Field Case Study: Obuasi, Ghana study site and design

The Obuasi Municipality District is one of thirty districts in the Ashanti Region of Ghana. Obuasi (latitude: 6º201N; longitude: 1º691W) is located in the southern Obuasi Municipal of the Ashanti Region of Ghana (Supplementary Figure 5A). Obuasi is bordered by Adansi South District in the east, the Amansie Central District in the west, Adansi North District in the north and Upper Denkyria district in the south. The AngloGold Ashanti (AGA) gold mining company conducted two rounds of indoor residual spraying since 2006 combined with larviciding in Obuasi, a largely urban area which has intense perennial malaria transmission. The AGA program also distributed insecticide-treated nets in local schools and hospitals and promoted increased use of artemisinin-based combination therapies at the company clinic and hospital. Within two years of initiating IRS and larviciding, the company hospital reported a 74% decline in malaria-related admissions and parasitaemia among school children in the area halved. Three *P. falciparum* prevalence surveys were conducted in the area, at baseline and post-intervention during which filter paper blood spots were collected.

1.1.3 Taq-Man Assay

The presence of an allele for a locus causes a change in melting temperature of an RT-PCR probe using high-resolution melting analysis. The 24-SNP molecular barcoding was performed using a 384-well format, as per (Daniels et al., 2008). Briefly for each reaction, template and water with a total volume of 2.5 µl was added to a 2.5 µl mix containing 0.125 µl 40×SNP assay and 2.5 µl Master Mix in a 384-well optical PCR plate and mixed, for a total reaction volume of 5 µl. The PCR plate was covered with an optical plate seal and amplified in an ABI 7900 HT. All isolates were then analysed using the Applied Biosystem's proprietary Allelic Discrimination and Absolute Quantitation software. If two alleles are found at a locus, with two melting peaks, the allele was defined as a mixed-allele call (MAC), indicating that there are multiple distinct parasite genomes present in the isolate. The multiplicity of infection was determined by counting the number of MACs per isolate haplotype and labelling monoclonal infections as isolates with MACs at less than or equal to 5% of SNP loci (i.e., 1 locus in the 24-SNP barcode) and multiclonal infections with MACs at greater than 5% of SNP loci per isolate (i.e., ≥ 2 loci in the 24-SNP barcode) (Rice et al., 2016).

1.2 Supplementary Results

1.2.1 Field Case Study: Obuasi, Ghana

A major motivation for genomic surveillance is to examine the effectiveness of interventions. SNP barcoding was performed on N=84 *P. falciparum* microscopic-positive isolates at a baseline study in 2006 (pre-IRS) and N=162 after two rounds of IRS in 2011 (post-IRS). Genotyping success was high for both surveys (≥75%, Supplementary Table 14). We found the number of MACs were high in both surveys (pre-IRS: 6 [3-10], post-IRS: 8 [3-13]), with even up to 23 of 24 SNP loci for an isolate having a MAC (Supplementary Figure 5B). The MAFs were below the 0.10 threshold in eight out of 24 loci (33.3%) in both surveys, resulting in only 17 SNPs to generate haplotypes (Supplementary Table 15). 78.0% of infections pre-IRS and 84.5% of infections post-IRS were defined as multiclonal infections (Supplementary Figure 5C). Given these results, haplotypes could only be created for 22% of isolates pre-IRS and 15.6% of isolates post-IRS to perform population genetics analyses, and for relatedness, structure or linkage analyses that require no missing data in the haplotype, only 7.3% of isolates pre-IRS and 9.9% of isolates post-IRS could be used (Supplementary Figure 7C). This led us to question the utility of this 24-SNP barcode on a broader scale in Africa and to further compare its performance to a larger barcode of 96 SNPs by a series of *in silico* experiments.

1.2.2 Detected polymorphisms of SNP molecular barcodes in African study populations

1.2.2.1 24-SNP barcode

The majority of loci (20/24) were biallelic in more than 70% of study populations, with one major and one minor allele per locus. Monoallelic loci are not informative as they lack variation to distinguish isolates from each other and therefore are likely heading to fixation if found predominantly in the population. Two loci were monoallelic in 73.3% (Pf3D7_13_v3_1429067) and 96.7% (Pf3D7_14_v3_755731) of study populations (Supplementary Figure 6A), as similarly observed in Eswatini, East Africa (Bei et al., 2018). Interestingly, Pf3D7_13_v3_1429067 was monoallelic in all West African countries but only in one study population in Central Africa (Kinshasa 2012, DRC) and East Africa (Kombewa 2014, Kenya), revealing spatial heterogeneity across regions in the level of fixation at this locus. There was one locus (Pf3D7_07_v3_1359804) found to be triallelic in 96.7% of study populations. This locus was typically found with one major allele ('A') and two minor alleles ('C'/'G') (Supplementary Figure 6A). There were two loci that were triallelic in only one population, Pf3D7_06_v3_937752 in Nzerekore, Guinea (2011) and Pf3D7_13_v3_158412 in Basse, Gambia (2013) (Supplementary Figure 6A). Monoallelic and triallelic loci found in >70% of the study populations (Supplementary Table 5) were removed from further analysis, resulting in a 20-SNP barcode (Supplementary Figure 1).

1.2.2.2 96-SNP barcode

85.6% (86/96) of loci were biallelic across the 30 study populations. Three loci were monoallelic in every population (Pf3D7_03_v3_707749, Pf3D7_04_v3_436931, Pf3D7_12_v3_1591956) and six other loci were monoallelic in >70% of study populations (Pf3D7_05_v3_720980, Pf3D7_06_v3_573735, Pf3D7_11_v3_306707, Pf3D7_11_v3_899285, Pf3D7_13_v3_619220, Pf3D7_14_v3_990734; Supplementary Figure 6B). Pf3D7_12_v3_1116284 was either triallelic or multiallelic in 73% of study populations and Pf3D7_03_v3_442947 was triallelic in three West African study populations (Cape-Coast 2014, Ghana, Nzerekore 2011, Guinea and Nioro du Sahel 2014, Mali) (Supplementary Figure 6B). The monoallelic, triallelic and multiallelic loci observed in >70% of the study populations (Supplementary Table 5) were also removed from downstream analysis, resulting in an 81-SNP barcode (Supplementary Figure 1).

2 Supplementary Figures and Tables

2.1 Supplementary Figures

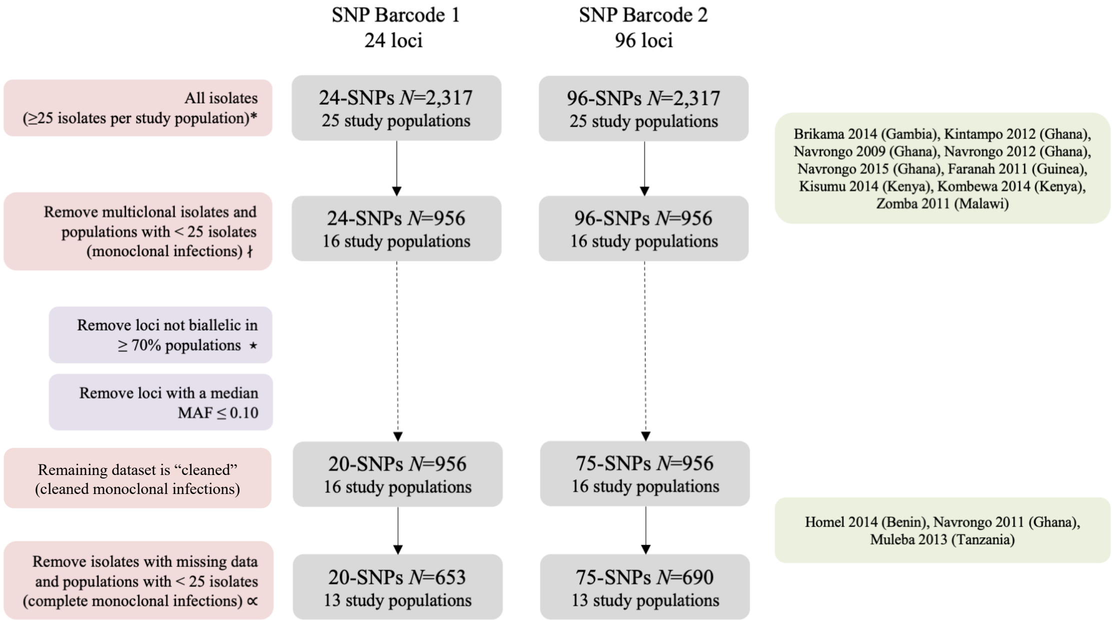

Supplementary Figure 1. Data used for genetic diversity and population genetics analyses. Red boxes describe the selection criteria for isolates in the data, purple boxes describe loci selection criteria and green boxes indicate the study populations that were removed to meet the criteria.

*Selected isolates in the MalariaGEN *Plasmodium falciparum* community Project version 6 obtained from African countries with moderate-to-high transmission and had more than 25 isolates per study population (study location by year).

⫳ Clonality based on the within-host inbreeding index (F_{WS}) using the whole-genome sequence calculated from the MalariaGEN database, where $F_{WS} < 0.95$ indicates multiclonal infections.

⋆ This includes loci that were monoallelic (one allele), triallelic (three alleles) and multiallelic (four alleles).

∝ Multilocus haplotypes were considered "complete" if there was an allele at each of the 20- or 75-SNP loci.

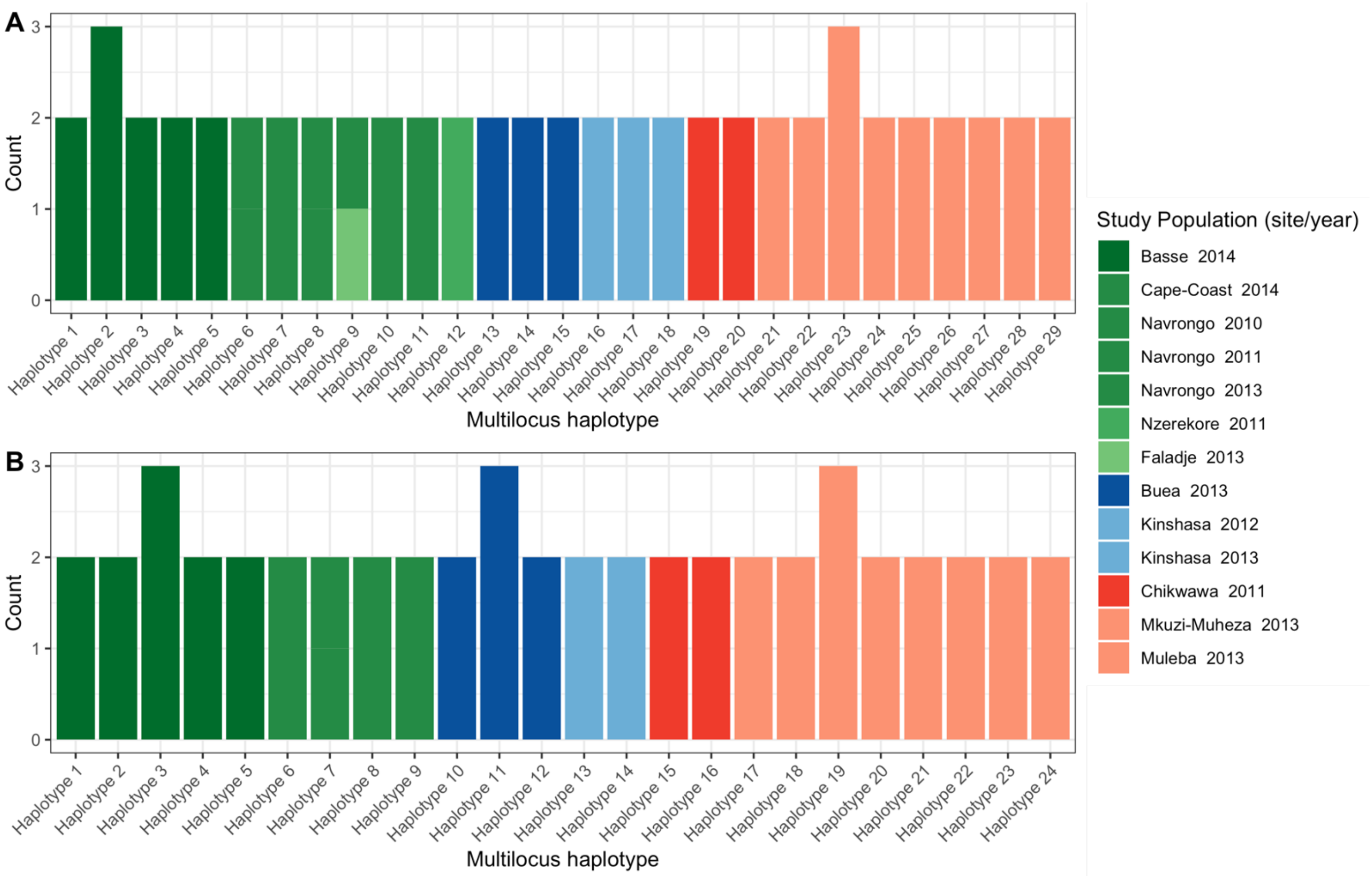

Supplementary Figure 2. Number of repeated multilocus haplotypes (count > 1) across 18 study populations for the **(A) 20-SNP** and **(B) 75-SNP barcode**. There were (A) *N*=29 and (B) *N*=24 repeated multilocus haplotypes in the 20- and 75-SNP barcodes respectively. Colours represent multilocus haplotypes found in study populations (study location by year) in West Africa (green hues), Central Africa (blue hues) and East Africa (red hues).

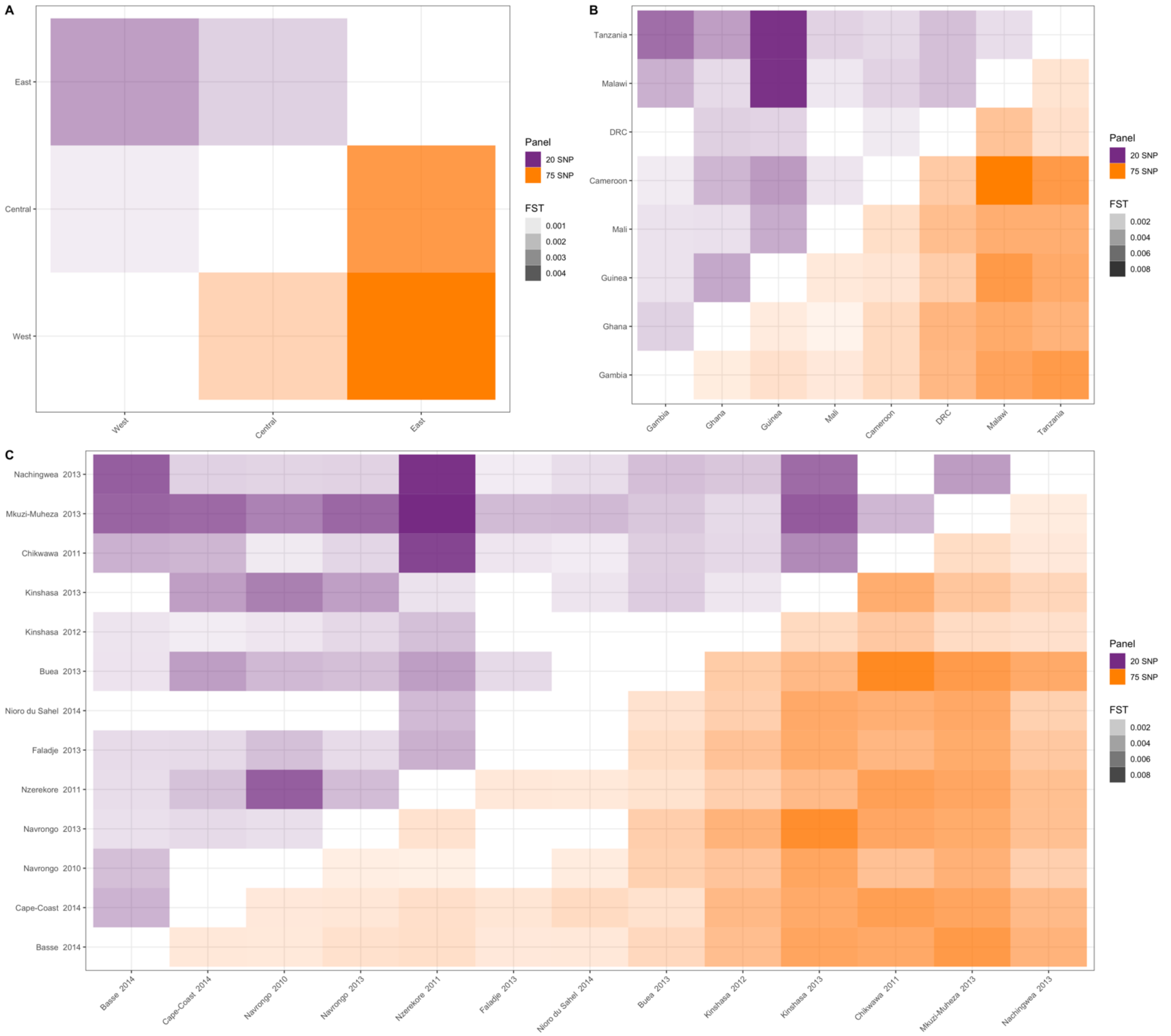

Supplementary Figure 3. Pairwise allelic differentiation for complete monoclonal infections using the 20-SNP and 75-SNP barcodes. *N*=653 and *N*=690 isolates for the 20- and 75-SNP barcodes respectively were used to calculate the pairwise F_{ST} values between West, Central and East Africa. F_{ST} was calculated as the proportion of allelic variance for the 20- and 75-SNP genotypes by **(A) 3 regions** (West, Central, East Africa), **(B) 8 countries**, and **(C) 13 study populations** (study location by year).

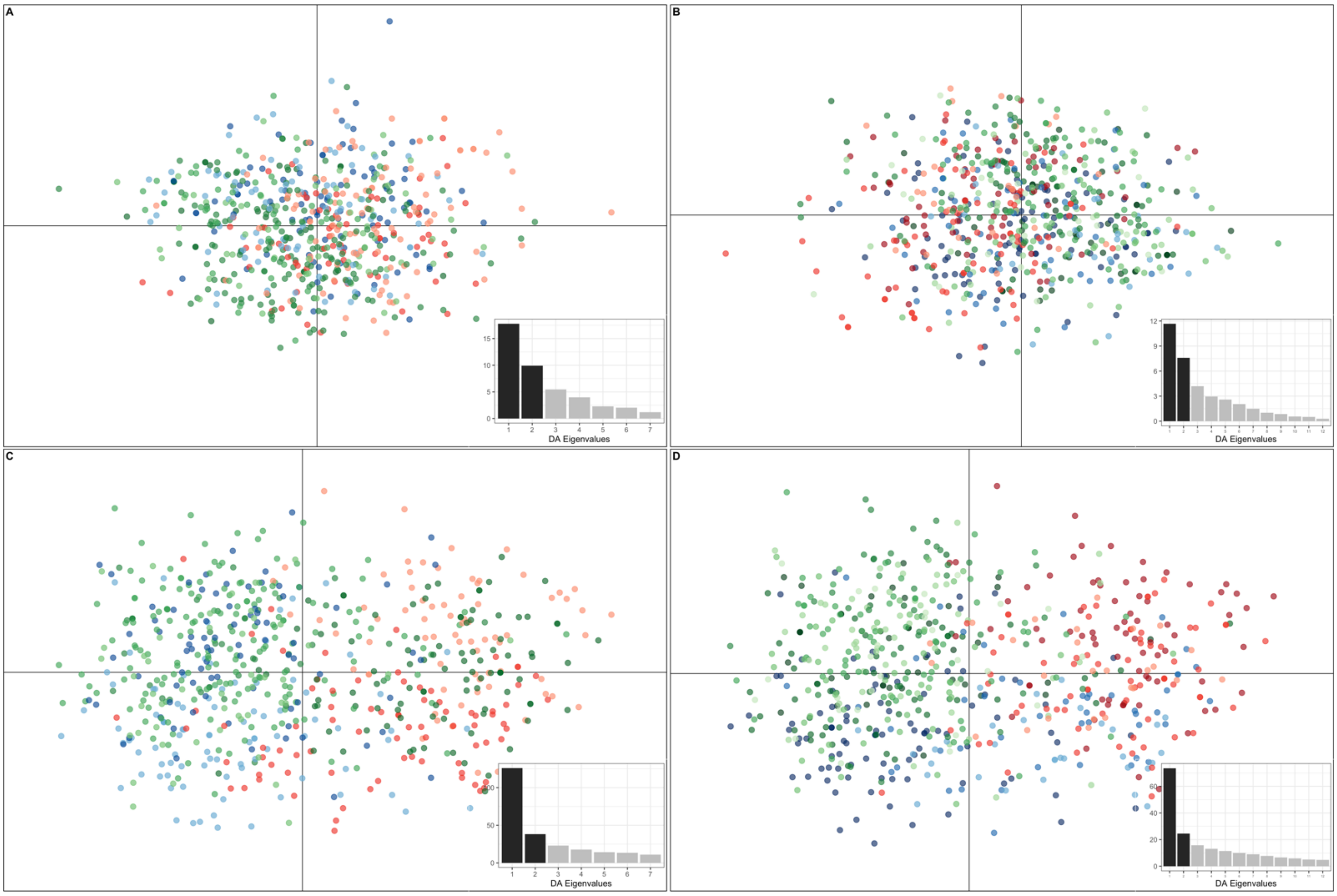

Supplementary Figure 4. Discriminant Analysis of Principal Components (DAPC) using the molecular barcodes at the **(A, C)** country level and **(B, D)** study population level. Using monoclonal infections with complete haplotypes and mixed-allele calls removed, **(A, B)** the 20-SNP panel (*N*=653) shows slight clustering and the **(C, D)** 75-SNP panel (*N*=690) generates clear clusters between countries and study populations in West Africa (green hues), Central Africa (blue hues) and East Africa (red hues). Note, the scree plot of eigenvalues is in the inset for each DAPC. Each dimension represents the two discriminant functions that explain the most variance in the original variables (dark grey bars).

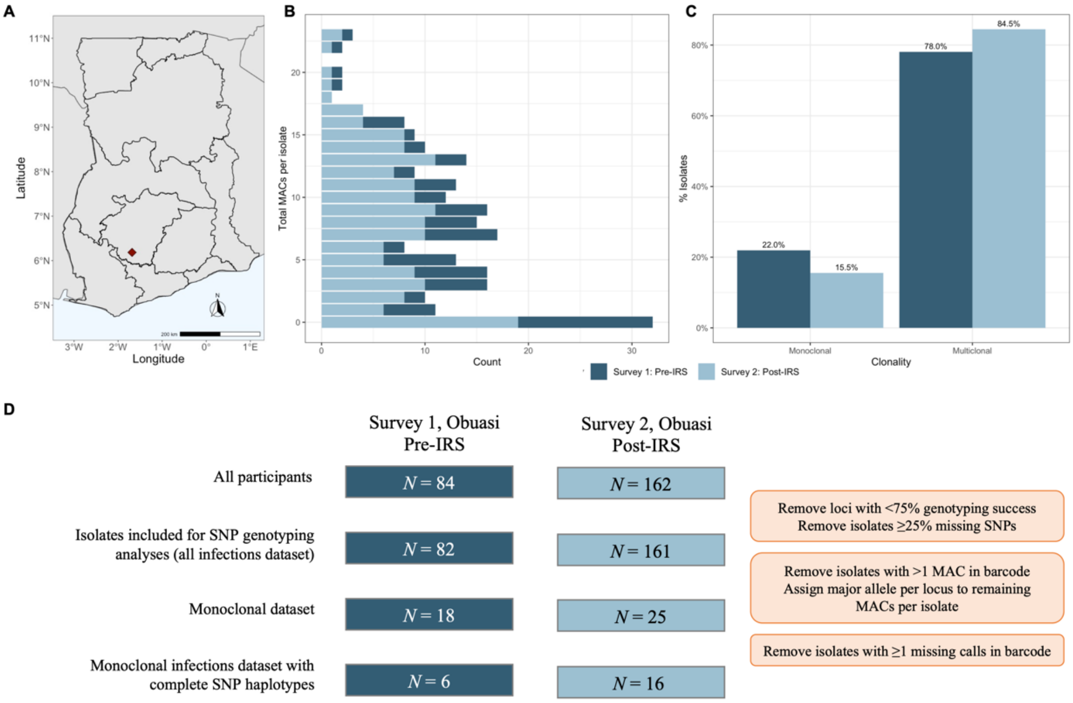

Supplementary Figure 5. Obuasi, Ghana study area and data breakdown. **(A)** Study Area: Obuasi (red diamond) is located in the Ashanti Region of Ghana. **(B)** Total number of mixed-allele calls (MACs) per isolate at the baseline survey before the IRS in 2006 (pre-IRS, dark blue) and after the cessation of the IRS programme in 2011 (post-IRS, light blue). **(C)** The percent of monoclonal and multiclonal infections pre- and post-IRS. **(D)** Data breakdown of isolates genotyped, found to be monoclonal and with complete infection haplotypes to be used for population genetics analysis (see Supplementary Material).

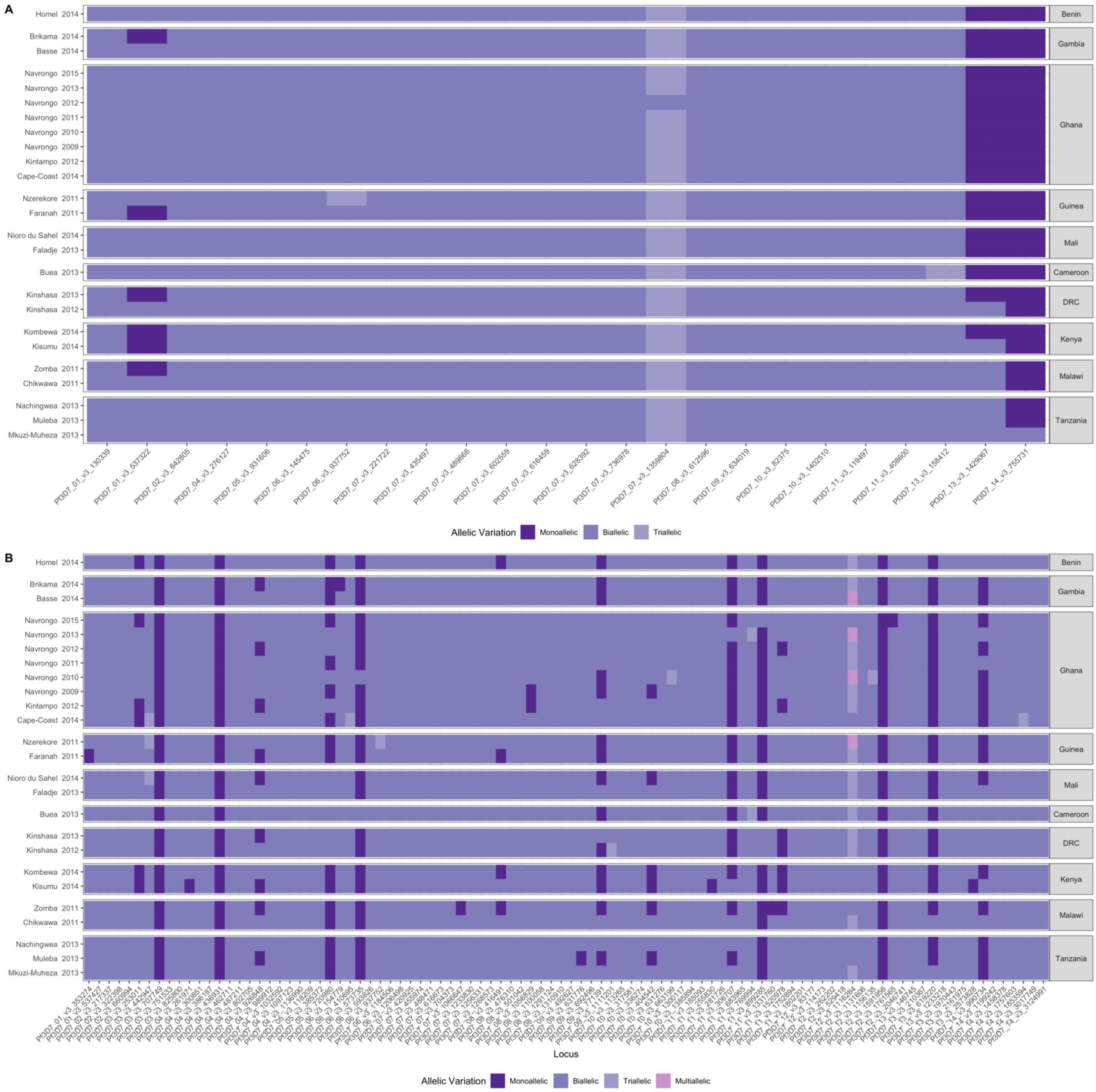

Supplementary Figure 6. Allelic variation per locus in the **(A)** 24 SNP and **(B)** 96 SNP barcode using monoclonal infections. The lightest hue of purple indicates a single allele call (monoallelic, i.e., A, C, G, or T), second-lightest purple hue indicates two allele calls for a locus (biallelic), the darkest hue of purple represents three allele calls per locus (triallelic), and pink represents four allele calls per locus (multiallelic). The grey panels on the right-hand side signify the countries where isolates were sampled, and the left-hand side shows the study population (study location by year) in that country.

2.2 Supplementary Tables

Supplementary Table 1. Relabelling SNP barcode identifications from referenced labels to positional information. The "old ID" refers to identifications outlined in papers for the 24-SNP (Daniels et al., 2008) and 96-SNP (Nkhoma et al., 2013) barcodes. The "new ID" column includes the *Plasmodium falciparum* 3D7 reference strain, chromosome the SNP locus is on, version of the 3D7 genome and position on the chromosome. Rows highlighted in grey signify where the two barcodes have the same SNP locus.

24-SNP barcode

Old ID	New ID
A1	Pf3D7_01_v3_130339
B1	Pf3D7_01_v3_537322
A2	Pf3D7_02_v3_842805
B2	Pf3D7_04_v3_276127
A3	Pf3D7_05_v3_931606
B3	Pf3D7_06_v3_145475
A4	Pf3D7_06_v3_937752
B4	Pf3D7_07_v3_221722
A5	Pf3D7_07_v3_435497
B5	Pf3D7_07_v3_489666
A6	Pf3D7_07_v3_602559
B6	Pf3D7_07_v3_616459
A7	Pf3D7_07_v3_628386
B7	Pf3D7_07_v3_736978
A8	Pf3D7_07_v3_1359804
B8	Pf3D7_08_v3_612596
A9	Pf3D7_09_v3_634019
B9	Pf3D7_10_v3_82375
A10	Pf3D7_10_v3_1402510
B10	Pf3D7_11_v3_119497
A11	Pf3D7_11_v3_408600
B11	Pf3D7_13_v3_158412
A12	Pf3D7_13_v3_1429067
B12	Pf3D7_14_v3_755731

96-SNP barcode (1/2)

Old ID	New ID
MAL01-354427	Pf3D7_01_v3_353274
MAL01-539149	Pf3D7_01_v3_537427
MAL02-217337	Pf3D7_02_v3_217337
MAL02-322398	Pf3D7_02_v3_322398
MAL02-660994	Pf3D7_02_v3_660994
MAL03-249686	Pf3D7_03_v3_253011
MAL03-439621	Pf3D7_03_v3_442947
MAL03-704420	Pf3D7_03_v3_707749
MAL03-748204	Pf3D7_03_v3_751533
MAL03-822470	Pf3D7_03_v3_825800
MAL04-268436	Pf3D7_04_v3_261971
MAL04-307321	Pf3D7_04_v3_300851
MAL04-393114	Pf3D7_04_v3_386187
MAL04-443828	Pf3D7_04_v3_436931
MAL04-469608	Pf3D7_04_v3_462711
MAL04-494108	Pf3D7_04_v3_487211
MAL04-809686	Pf3D7_04_v3_802705
MAL04-933828	Pf3D7_04_v3_866848
MAL04-994352	Pf3D7_04_v3_989912
MAL04-1037032	Pf3D7_04_v3_1032586
MAL04-1101563	Pf3D7_04_v3_1097123
MAL04-1140617	Pf3D7_04_v3_1136990
MAL05-118209	Pf3D7_05_v3_118209
MAL05-385372	Pf3D7_05_v3_385372
MAL05-720978	Pf3D7_05_v3_720980
MAL06-154776	Pf3D7_06_v3_154779
MAL06-410691	Pf3D7_06_v3_410695
MAL06-573730	Pf3D7_06_v3_573735
MAL06-593821	Pf3D7_06_v3_593826
MAL06-937750	Pf3D7_06_v3_937752
MAL06-1184507	Pf3D7_06_v3_1184506
MAL06-1206499	Pf3D7_06_v3_1206498
MAL07-476303	Pf3D7_07_v3_420865
MAL07-511352	Pf3D7_07_v3_455974
MAL07-543849	Pf3D7_07_v3_488471
MAL07-672051	Pf3D7_07_v3_616673
MAL07-759749	Pf3D7_07_v3_704373
MAL07-1122023	Pf3D7_07_v3_1066647
MAL07-1288606	Pf3D7_07_v3_1233830
MAL07-1311707	Pf3D7_07_v3_1256331
MAL07-1323949	Pf3D7_07_v3_1268573
MAL08-417613	Pf3D7_08_v3_416491
MAL08-477431	Pf3D7_08_v3_476310
MAL08-502163	Pf3D7_08_v3_501042
MAL08-1057901	Pf3D7_08_v3_1056829
MAL08-1101130	Pf3D7_08_v3_1100058
MAL08-1286195	Pf3D7_08_v3_1291124
MAL08-1311981	Pf3D7_08_v3_1310910

96-SNP barcode (2/2)

Old ID	New ID
MAL09-486618	Pf3D7_09_v3_486627
MAL09-631767	Pf3D7_09_v3_631776
MAL09-686487	Pf3D7_09_v3_686496
MAL09-771381	Pf3D7_09_v3_771391
MAL09-1111191	Pf3D7_09_v3_1111201
MAL10-113266	Pf3D7_10_v3_113265
MAL10-317580	Pf3D7_10_v3_317581
MAL10-336274	Pf3D7_10_v3_336274
MAL10-404541	Pf3D7_10_v3_404542
MAL10-631275	Pf3D7_10_v3_631276
MAL10-663198	Pf3D7_10_v3_663199
MAL10-1300115	Pf3D7_10_v3_1300117
MAL10-1385891	Pf3D7_10_v3_1385894
MAL11-185094	Pf3D7_11_v3_185093
MAL11-255830	Pf3D7_11_v3_255830
MAL11-281726	Pf3D7_11_v3_281726
MAL11-304323	Pf3D7_11_v3_306707
MAL11-683964	Pf3D7_11_v3_683965
MAL11-769993	Pf3D7_11_v3_769994
MAL11-896199	Pf3D7_11_v3_898685
MAL11-1331758	Pf3D7_11_v3_1331760
MAL11-1520975	Pf3D7_11_v3_1520978
MAL11-1749804	Pf3D7_11_v3_1752894
MAL11-1802198	Pf3D7_11_v3_1802201
MAL12-83177	Pf3D7_12_v3_83177
MAL12-114173	Pf3D7_12_v3_114173
MAL12-262199	Pf3D7_12_v3_262202
MAL12-529412	Pf3D7_12_v3_529418
MAL12-1116275	Pf3D7_12_v3_1116284
MAL12-1131597	Pf3D7_12_v3_1131606
MAL12-1156125	Pf3D7_12_v3_1156135
MAL12-1591944	Pf3D7_12_v3_1591956
MAL12-1761553	Pf3D7_12_v3_1761565
MAL12-2046727	Pf3D7_12_v3_2046741
MAL13-146947	Pf3D7_13_v3_146745
MAL13-210569	Pf3D7_13_v3_210367
MAL13-619422	Pf3D7_13_v3_619220
MAL13-1233419	Pf3D7_13_v3_1233218
MAL13-1966774	Pf3D7_13_v3_1970443
MAL13-2158308	Pf3D7_13_v3_2161975
MAL13-2570163	Pf3D7_13_v3_2573828
MAL14-990875	Pf3D7_14_v3_990734
MAL14-1199184	Pf3D7_14_v3_1198642
MAL14-1458321	Pf3D7_14_v3_1458378
MAL14-1757546	Pf3D7_14_v3_1757603
MAL14-1853537	Pf3D7_14_v3_1853594
MAL14-3017684	Pf3D7_14_v3_3017749
MAL14-3124866	Pf3D7_14_v3_3124991

Supplementary Table 2. Minor allele frequencies (MAFs) for each study population using all infections for the 24-SNP barcode. Accompanying file: .xls file.

Supplementary Table 3. Minor allele frequencies (MAFs) for each study population using all infections for the 96-SNP barcode. Accompanying file: .xls file.

Supplementary Table 4. Clonality of infections as calculated by F_{WS} and the number of mixed-allele calls (MAC) per locus for the 24-SNP and 96-SNP barcodes. Infections are classified as "monoclonal" when $F_{WS} \geq 0.95$ and "multiclonal" when $F_{WS} < 0.95$. If more than 5% of the 24- and 96-SNP barcodes contain MACs (i.e., MAC > 1 or MAC > 5, respectively), then the infection would be labelled as a monoclonal infection and vice versa.

Country	Study Location	Year	*N*	Monoclonal ($F_{WS} \geq 0.95$)	Multiclonal (F_{WS}<0.95)	24 SNP Barcode		96 SNP Barcode	
						MAC ≤ 1	MAC > 1	MAC ≤ 5	MAC > 5
Benin	Homel	2014	36	25 (69.4)	11 (30.6)	24 (66.7)	12 (33.3)	23 (63.9)	13 (36.1)
Gambia	Basse	2014	81	51 (63)	30 (37)	50 (61.7)	31 (38.3)	48 (59.3)	33 (40.7)
	Brikama	2014	42	17 (40.5)	25 (59.5)	19 (45.2)	23 (54.8)	17 (40.5)	25 (59.5)
Ghana	Cape-Coast	2014	100	58 (58)	42 (42)	57 (57)	43 (43)	55 (55)	45 (45)
	Kintampo	2012	35	17 (48.6)	18 (51.4)	16 (45.7)	19 (54.3)	16 (45.7)	19 (54.3)
	Navrongo	2009	46	20 (43.5)	26 (56.5)	20 (43.5)	26 (56.5)	18 (39.1)	28 (60.9)
	Navrongo	2010	135	69 (51.1)	66 (48.9)	67 (49.6)	68 (50.4)	63 (46.7)	72 (53.3)
	Navrongo	2011	93	39 (41.9)	54 (58.1)	37 (39.8)	56 (60.2)	32 (34.4)	61 (65.6)
	Navrongo	2012	39	21 (53.8)	18 (46.2)	21 (53.8)	18 (46.2)	19 (48.7)	20 (51.3)
	Navrongo	2013	241	86 (35.7)	155 (64.3)	87 (36.1)	154 (63.9)	82 (34)	159 (66)
	Navrongo	2015	57	24 (42.1)	33 (57.9)	23 (40.4)	34 (59.6)	24 (42.1)	33 (57.9)
Guinea	Faranah	2011	37	15 (40.5)	22 (59.5)	15 (40.5)	22 (59.5)	10 (27)	27 (73)
	Nzerekore	2011	112	59 (52.7)	53 (47.3)	57 (50.9)	55 (49.1)	56 (50)	56 (50)
Mali	Faladje	2013	124	62 (50)	62 (50)	61 (49.2)	63 (50.8)	57 (46)	67 (54)
	Nioro du Sahel	2014	49	31 (63.3)	18 (36.7)	32 (65.3)	17 (34.7)	30 (61.2)	19 (38.8)
Cameroon	Buea	2013	235	116 (49.4)	119 (50.6)	112 (47.7)	123 (52.3)	106 (45.1)	129 (54.9)
DRC	Kinshasa	2012	171	73 (42.7)	98 (57.3)	72 (42.1)	99 (57.9)	62 (36.3)	109 (63.7)
	Kinshasa	2013	108	49 (45.4)	59 (54.6)	46 (42.6)	62 (57.4)	44 (40.7)	64 (59.3)
Kenya	Kisumu	2014	34	10 (29.4)	24 (70.6)	10 (29.4)	24 (70.6)	8 (23.5)	26 (76.5)
	Kombewa	2014	26	11 (42.3)	15 (57.7)	11 (42.3)	15 (57.7)	9 (34.6)	17 (65.4)
Malawi	Chikwawa	2011	221	88 (39.8)	133 (60.2)	88 (39.8)	133 (60.2)	81 (36.7)	140 (63.3)
	Zomba	2011	33	14 (42.4)	19 (57.6)	14 (42.4)	19 (57.6)	13 (39.4)	20 (60.6)
Tanzania	Mkuzi-Muheza	2013	145	91 (62.8)	54 (37.2)	91 (62.8)	54 (37.2)	85 (58.6)	60 (41.4)
	Muleba	2013	52	27 (51.9)	25 (48.1)	26 (50)	26 (50)	25 (48.1)	27 (51.9)
	Nachingwea	2013	65	32 (49.2)	33 (50.8)	32 (49.2)	33 (50.8)	28 (43.1)	37 (56.9)
		Total	2,317	1,105 (47.7)	1,212 (52.3)	1,088 (47.0)	1,229 (53.0)	1,011 (43.6)	1,306 (56.4)

Data reflect the No. (% [n/N]) of *P. falciparum* genotypes.
N represents the number of isolates, and *n* represents the number of isolates for a given variable.

Supplementary Table 5. Biallelic loci across each SNP barcode in the 25 study populations. Loci where the majority were not biallelic in 20 or more populations were removed from population genetics analyses (highlighted in grey).

24-SNP Barcode		96-SNP barcode			
**Locus**	**Biallelic (%)**	**Locus**	**Biallelic (%)**	**Locus**	**Biallelic (%)**
Pf3D7_01_v3_130339	25 (100)	Pf3D7_01_v3_353274	24 (96)	Pf3D7_09_v3_492627	25 (100)
Pf3D7_01_v3_537322	19 (76)	Pf3D7_01_v3_537427	25 (100)	Pf3D7_09_v3_631776	24 (96)
Pf3D7_02_v3_842805	25 (100)	Pf3D7_02_v3_217337	25 (100)	Pf3D7_09_v3_692496	25 (100)
Pf3D7_04_v3_276127	25 (100)	Pf3D7_02_v3_322398	25 (100)	Pf3D7_09_v3_771391	11 (44)
Pf3D7_05_v3_931606	25 (100)	Pf3D7_02_v3_660994	25 (100)	Pf3D7_09_v3_1111201	24 (96)
Pf3D7_06_v3_145475	25 (100)	Pf3D7_03_v3_253011	19 (76)	Pf3D7_10_v3_113265	25 (100)
Pf3D7_06_v3_937752	24 (96)	Pf3D7_03_v3_442947	22 (88)	Pf3D7_10_v3_317581	25 (100)
Pf3D7_07_v3_221722	25 (100)	Pf3D7_03_v3_707749	0 (0)	Pf3D7_10_v3_336274	25 (100)
Pf3D7_07_v3_435497	25 (100)	Pf3D7_03_v3_751533	25 (100)	Pf3D7_10_v3_404542	19 (76)
Pf3D7_07_v3_489666	25 (100)	Pf3D7_03_v3_825800	25 (100)	Pf3D7_10_v3_631276	25 (100)
Pf3D7_07_v3_602559	25 (100)	Pf3D7_04_v3_261971	24 (96)	Pf3D7_10_v3_663199	24 (96)
Pf3D7_07_v3_616459	25 (100)	Pf3D7_04_v3_300851	25 (100)	Pf3D7_10_v3_1300117	25 (100)
Pf3D7_07_v3_628392	25 (100)	Pf3D7_04_v3_386187	25 (100)	Pf3D7_10_v3_1385894	25 (100)
Pf3D7_07_v3_736978	25 (100)	Pf3D7_04_v3_436931	0 (0)	Pf3D7_11_v3_185093	25 (100)
Pf3D7_07_v3_1359804	1 (4)	Pf3D7_04_v3_462711	25 (100)	Pf3D7_11_v3_255830	24 (96)
Pf3D7_08_v3_612596	25 (100)	Pf3D7_04_v3_487211	25 (100)	Pf3D7_11_v3_281726	25 (100)
Pf3D7_09_v3_634019	25 (100)	Pf3D7_04_v3_802705	25 (100)	Pf3D7_11_v3_306707	5 (20)
Pf3D7_10_v3_82375	25 (100)	Pf3D7_04_v3_926848	16 (64)	Pf3D7_11_v3_683965	25 (100)
Pf3D7_10_v3_1402510	25 (100)	Pf3D7_04_v3_989912	25 (100)	Pf3D7_11_v3_769994	23 (92)
Pf3D7_11_v3_119497	25 (100)	Pf3D7_04_v3_1032592	25 (100)	Pf3D7_11_v3_899285	3 (12)
Pf3D7_11_v3_408600	25 (100)	Pf3D7_04_v3_1097123	25 (100)	Pf3D7_11_v3_1331760	24 (96)
Pf3D7_13_v3_158412	24 (96)	Pf3D7_04_v3_1136990	25 (100)	Pf3D7_11_v3_1520978	18 (72)
Pf3D7_13_v3_1429067	7 (28)	Pf3D7_05_v3_118209	25 (100)	Pf3D7_11_v3_1752894	25 (100)
Pf3D7_14_v3_755731	1 (4)	Pf3D7_05_v3_385372	25 (100)	Pf3D7_11_v3_1802201	25 (100)
		Pf3D7_05_v3_720980	9 (36)	Pf3D7_12_v3_83177	25 (100)
		Pf3D7_06_v3_154779	24 (96)	Pf3D7_12_v3_114173	25 (100)
		Pf3D7_06_v3_410695	24 (96)	Pf3D7_12_v3_262202	25 (100)
		Pf3D7_06_v3_573735	2 (8)	Pf3D7_12_v3_529418	25 (100)
		Pf3D7_06_v3_593826	25 (100)	Pf3D7_12_v3_1116284	7 (28)
		Pf3D7_06_v3_937752	24 (96)	Pf3D7_12_v3_1131606	25 (100)
		Pf3D7_06_v3_1184506	25 (100)	Pf3D7_12_v3_1156135	24 (96)
		Pf3D7_06_v3_1206498	25 (100)	Pf3D7_12_v3_1591956	0 (0)
		Pf3D7_07_v3_420925	25 (100)	Pf3D7_12_v3_1761565	24 (96)
		Pf3D7_07_v3_455974	25 (100)	Pf3D7_12_v3_2046741	25 (100)
		Pf3D7_07_v3_488471	25 (100)	Pf3D7_13_v3_146745	25 (100)
		Pf3D7_07_v3_616673	25 (100)	Pf3D7_13_v3_210367	25 (100)
		Pf3D7_07_v3_704373	25 (100)	Pf3D7_13_v3_619220	2 (8)
		Pf3D7_07_v3_1066647	24 (96)	Pf3D7_13_v3_1233218	25 (100)
		Pf3D7_07_v3_1233830	25 (100)	Pf3D7_13_v3_1970443	25 (100)
		Pf3D7_07_v3_1256331	25 (100)	Pf3D7_13_v3_2161975	25 (100)
		Pf3D7_07_v3_1268573	25 (100)	Pf3D7_13_v3_2573828	24 (96)
		Pf3D7_08_v3_416491	21 (84)	Pf3D7_14_v3_990734	9 (36)
		Pf3D7_08_v3_476310	25 (100)	Pf3D7_14_v3_1199242	25 (100)
		Pf3D7_08_v3_501042	25 (100)	Pf3D7_14_v3_1458378	25 (100)
		Pf3D7_08_v3_1056829	23 (92)	Pf3D7_14_v3_1757603	25 (100)
		Pf3D7_08_v3_1100058	25 (100)	Pf3D7_14_v3_1853594	24 (96)
		Pf3D7_08_v3_1291124	25 (100)	Pf3D7_14_v3_3017749	25 (100)
		Pf3D7_08_v3_1310910	25 (100)	Pf3D7_14_v3_3124991	25 (100)

Data reflect No. (%[n/N]).

Supplementary Table 6. Minor allele frequency (MAF) for each locus in the 20-SNP and 81-SNP barcodes. Loci highlighted in grey indicate that the median MAF was below 0.10 the majority and were removed from population genetics analyses.

20-SNP Barcode		81-SNP barcode			
Locus	MAF (median [IQR])	Locus	MAF (median [IQR])	Locus	MAF (median [IQR])
Pf3D7_01_v3_130339	0.320 [0.236 - 0.354]	Pf3D7_01_v3_353274	0.252 [0.225 - 0.285]	Pf3D7_08_v3_1310910	0.430 [0.388 - 0.448]
Pf3D7_02_v3_842805	0.307 [0.292 - 0.373]	Pf3D7_01_v3_537427	0.404 [0.340 - 0.477]	Pf3D7_09_v3_492627	0.254 [0.236 - 0.297]
Pf3D7_04_v3_276127	0.158 [0.126 - 0.197]	Pf3D7_02_v3_217337	0.244 [0.188 - 0.289]	Pf3D7_09_v3_631776	0.095 [0.077 - 0.119]
Pf3D7_05_v3_931606	0.269 [0.215 - 0.304]	Pf3D7_02_v3_322398	0.272 [0.224 - 0.297]	Pf3D7_09_v3_692496	0.082 [0.068 - 0.094]
Pf3D7_06_v3_145475	0.374 [0.323 - 0.443]	Pf3D7_02_v3_660994	0.242 [0.202 - 0.285]	Pf3D7_09_v3_1111201	0.338 [0.310 - 0.371]
Pf3D7_06_v3_937752	0.403 [0.384 - 0.436]	Pf3D7_03_v3_442947	0.180 [0.150 - 0.208]	Pf3D7_10_v3_113265	0.380 [0.325 - 0.444]
Pf3D7_07_v3_221722	0.133 [0.096 - 0.150]	Pf3D7_03_v3_751533	0.187 [0.170 - 0.251]	Pf3D7_10_v3_317581	0.455 [0.397 - 0.471]
Pf3D7_07_v3_435497	0.351 [0.292 - 0.418]	Pf3D7_03_v3_825800	0.214 [0.171 - 0.256]	Pf3D7_10_v3_336274	0.196 [0.144 - 0.245]
Pf3D7_07_v3_489666	0.423 [0.409 - 0.449]	Pf3D7_04_v3_261971	0.196 [0.156 - 0.240]	Pf3D7_10_v3_631276	0.329 [0.290 - 0.361]
Pf3D7_07_v3_602559	0.375 [0.339 - 0.432]	Pf3D7_04_v3_300851	0.202 [0.153 - 0.266]	Pf3D7_10_v3_663199	0.171 [0.143 - 0.224]
Pf3D7_07_v3_616459	0.414 [0.371 - 0.470]	Pf3D7_04_v3_386187	0.192 [0.128 - 0.220]	Pf3D7_10_v3_1300117	0.436 [0.408 - 0.481]
Pf3D7_07_v3_628392	0.370 [0.352 - 0.439]	Pf3D7_04_v3_462711	0.401 [0.376 - 0.456]	Pf3D7_10_v3_1385894	0.298 [0.237 - 0.323]
Pf3D7_07_v3_736978	0.268 [0.226 - 0.324]	Pf3D7_04_v3_487211	0.275 [0.244 - 0.329]	Pf3D7_11_v3_185093	0.372 [0.346 - 0.447]
Pf3D7_08_v3_612596	0.260 [0.220 - 0.281]	Pf3D7_04_v3_802705	0.330 [0.285 - 0.371]	Pf3D7_11_v3_255830	0.084 [0.050 - 0.099]
Pf3D7_09_v3_634019	0.389 [0.356 - 0.424]	Pf3D7_04_v3_989912	0.425 [0.393 - 0.439]	Pf3D7_11_v3_281726	0.272 [0.231 - 0.322]
Pf3D7_10_v3_82375	0.436 [0.407 - 0.450]	Pf3D7_04_v3_1032592	0.368 [0.349 - 0.434]	Pf3D7_11_v3_683965	0.201 [0.149 - 0.246]
Pf3D7_10_v3_1402510	0.315 [0.270 - 0.382]	Pf3D7_04_v3_1097123	0.386 [0.364 - 0.449]	Pf3D7_11_v3_769994	0.276 [0.220 - 0.340]
Pf3D7_11_v3_119497	0.172 [0.139 - 0.228]	Pf3D7_04_v3_1136990	0.380 [0.292 - 0.408]	Pf3D7_11_v3_1331760	0.052 [0.040 - 0.065]
Pf3D7_11_v3_408600	0.426 [0.402 - 0.470]	Pf3D7_05_v3_118209	0.392 [0.328 - 0.459]	Pf3D7_11_v3_1752894	0.400 [0.376 - 0.440]
Pf3D7_13_v3_158412	0.440 [0.413 - 0.466]	Pf3D7_05_v3_385372	0.408 [0.375 - 0.472]	Pf3D7_11_v3_1802201	0.199 [0.162 - 0.238]
Pf3D7_01_v3_130339	0.320 [0.236 - 0.354]	Pf3D7_06_v3_154779	0.174 [0.154 - 0.201]	Pf3D7_12_v3_83177	0.444 [0.400 - 0.471]
		Pf3D7_06_v3_410695	0.272 [0.233 - 0.300]	Pf3D7_12_v3_114173	0.442 [0.392 - 0.454]
		Pf3D7_06_v3_593826	0.333 [0.303 - 0.384]	Pf3D7_12_v3_262202	0.254 [0.193 - 0.303]
		Pf3D7_06_v3_937752	0.402 [0.382 - 0.428]	Pf3D7_12_v3_529418	0.434 [0.415 - 0.471]
		Pf3D7_06_v3_1184506	0.394 [0.346 - 0.451]	Pf3D7_12_v3_1131606	0.403 [0.327 - 0.424]
		Pf3D7_06_v3_1206498	0.348 [0.299 - 0.393]	Pf3D7_12_v3_1156135	0.454 [0.387 - 0.480]
		Pf3D7_07_v3_420925	0.374 [0.339 - 0.440]	Pf3D7_12_v3_1761565	0.141 [0.079 - 0.166]
		Pf3D7_07_v3_455974	0.420 [0.359 - 0.460]	Pf3D7_12_v3_2046741	0.348 [0.333 - 0.426]
		Pf3D7_07_v3_488471	0.433 [0.394 - 0.470]	Pf3D7_13_v3_146745	0.388 [0.358 - 0.440]
		Pf3D7_07_v3_616673	0.256 [0.228 - 0.271]	Pf3D7_13_v3_210367	0.406 [0.338 - 0.430]
		Pf3D7_07_v3_704373	0.393 [0.342 - 0.443]	Pf3D7_13_v3_1233218	0.380 [0.333 - 0.425]
		Pf3D7_07_v3_1066647	0.169 [0.127 - 0.199]	Pf3D7_13_v3_1970443	0.424 [0.370 - 0.470]
		Pf3D7_07_v3_1233830	0.422 [0.386 - 0.444]	Pf3D7_13_v3_2161975	0.334 [0.298 - 0.373]
		Pf3D7_07_v3_1256331	0.178 [0.150 - 0.204]	Pf3D7_13_v3_2573828	0.186 [0.143 - 0.205]
		Pf3D7_07_v3_1268573	0.362 [0.333 - 0.434]	Pf3D7_14_v3_1199242	0.366 [0.300 - 0.414]
		Pf3D7_08_v3_416491	0.062 [0.036 - 0.109]	Pf3D7_14_v3_1458378	0.432 [0.364 - 0.472]
		Pf3D7_08_v3_476310	0.426 [0.371 - 0.481]	Pf3D7_14_v3_1757603	0.370 [0.281 - 0.424]
		Pf3D7_08_v3_501042	0.349 [0.265 - 0.412]	Pf3D7_14_v3_1853594	0.265 [0.228 - 0.310]
		Pf3D7_08_v3_1056829	0.092 [0.061 - 0.166]	Pf3D7_14_v3_3017749	0.404 [0.382 - 0.452]
		Pf3D7_08_v3_1100058	0.455 [0.397 - 0.468]	Pf3D7_14_v3_3124991	0.261 [0.220 - 0.294]
		Pf3D7_08_v3_1291124	0.334 [0.306 - 0.422]		

IQR = Interquartile Range.

Supplementary Table 7. Multilocus linkage disequilibrium ($\bar{r}_d$) calculated using "complete monoclonal infections" dataset in the total population and across the regional, country and study population levels.

	20-SNP barcode		75-SNP barcode	
Study population	*N*	$\bar{r}_d$ (*p*-value)	*N*	$\bar{r}_d$ (*p*-value)
Total	653	0.0005 (0.171)	690	0.0002 (0.055)
**West Africa**	**306**	**0.0025 (0.004)***	**345**	**0.0002 (0.206)**
**The Gambia**	**39**	**0.0095 (0.011)***	**41**	**0.0071 (0.001)***
Basse 2014	39	0.0095 (0.011)*	41	0.0071 (0.001)*
**Ghana**	**156**	**0.0039 (0.004)***	**175**	**0.0003 (0.184)**
Cape-Coast 2014	48	0.0059 (0.022)*	55	-0.0006 (0.787)
Navrongo 2010	45	0.0027 (0.186)	49	0.0014 (0.038)*
Navrongo 2013	63	0.0061 (0.017)*	71	0.0015 (0.019)*
**Guinea**	**46**	**0.0009 (0.361)**	**48**	**0.0012 (0.062)***
Nzerekore 2011	46	0.0009 (0.359)	48	0.0012 (0.066)
**Mali**	**65**	**0.0007 (0.353)**	**81**	**0.0006 (0.124)**
Faladje 2013	40	-0.0003 (0.521)	52	0.0005 (0.235)
Nioro du Sahel 2014	25	0.0020 (0.302)	29	0.0008 (0.239)
**Central Africa**	**177**	**-0.0019 (0.960)**	**173**	**0.0005 (0.100)**
**Cameroon**	**91**	**-0.0011 (0.710)**	**92**	**0.0005 (0.184)**
Buea 2013	91	-0.0011 (0.724)	92	0.0005 (0.178)
**DRC**	**86**	**-0.0016 (0.792)**	**81**	**0.0005 (0.172)**
Kinshasa 2012	54	<0.0001 (0.457)	49	0.0004 (0.268)
Kinshasa 2013	32	-0.0052 (0.908)	32	0.0026 (0.016)*
**East Africa**	**170**	**0.0006 (0.297)**	**172**	**0.0008 (0.016)***
**Malawi**	**69**	**0.0011 (0.306)**	**74**	**0.0009 (0.094)**
Chikwawa 2011	69	0.0011 (0.328)	74	0.0009 (0.093)
**Tanzania**	**101**	**0.0019 (0.101)**	**98**	**0.0017 (0.001)***
Mkuzi-Muheza 2013	74	0.0014 (0.236)	72	0.0033 (0.001)*
Nachingwea 2013	27	0.0012 (0.399)	26	0.0003 (0.387)

$\bar{r}_d$ = standardised index of association
N = number of isolates with complete monoclonal infections (no missing data)
p-values are indicated in the brackets with * to denote a significant value < 0.05.

Supplementary Table 8. Pairwise multilocus linkage disequilibrium ($\bar{r}_d$) calculated using the "complete monoclonal infections" dataset ($F_{WS} \geq 0.95$) for each study population for the 20-SNP barcode. Accompanying file: .xls

Supplementary Table 9. Pairwise multilocus linkage disequilibrium ($\bar{r}_d$) calculated using the "complete monoclonal infections" dataset ($F_{WS} \geq 0.95$) for each study population for the 75-SNP barcode. Accompanying file: .xls

Supplementary Table 10. Genetic differentiation of *P. falciparum* populations by locus for the “complete monoclonal infections” dataset for the 20-SNP barcode and 75-SNP barcode.

20-SNP		75-SNP			
**Locus**	F_{ST}	**Locus**	F_{ST}	**Locus**	F_{ST}
Pf3D7_01_v3_130339	0	Pf3D7_01_v3_353274	0	Pf3D7_08_v3_1291124	0.00090
Pf3D7_02_v3_842805	0.00010	Pf3D7_01_v3_537427	0.00370	Pf3D7_08_v3_1310910	0
Pf3D7_04_v3_276127	0.00040	Pf3D7_02_v3_217337	0	Pf3D7_09_v3_492627	0.00200
Pf3D7_05_v3_931606	0.00190	Pf3D7_02_v3_322398	0	Pf3D7_09_v3_1111201	0.00110
Pf3D7_06_v3_145475	0.00100	Pf3D7_02_v3_660994	0.00150	Pf3D7_10_v3_113265	0.00960
Pf3D7_06_v3_937752	0	Pf3D7_03_v3_442947	0.00010	Pf3D7_10_v3_317581	0.00100
Pf3D7_07_v3_221722	0	Pf3D7_03_v3_751533	0.00050	Pf3D7_10_v3_336274	0.00040
Pf3D7_07_v3_435497	0.00640	Pf3D7_03_v3_825800	0.00070	Pf3D7_10_v3_631276	0
Pf3D7_07_v3_489666	0.00040	Pf3D7_04_v3_261971	0	Pf3D7_10_v3_663199	0.06360
Pf3D7_07_v3_602559	0	Pf3D7_04_v3_300851	0.00140	Pf3D7_10_v3_1300117	0.00110
Pf3D7_07_v3_616459	0.00160	Pf3D7_04_v3_386187	0	Pf3D7_10_v3_1385894	0.00030
Pf3D7_07_v3_628392	0.00000	Pf3D7_04_v3_462711	0.00090	Pf3D7_11_v3_185093	0
Pf3D7_07_v3_736978	0.00020	Pf3D7_04_v3_487211	0	Pf3D7_11_v3_281726	0
Pf3D7_08_v3_612596	0.00390	Pf3D7_04_v3_802705	0.02510	Pf3D7_11_v3_683965	0.00050
Pf3D7_09_v3_634019	0.00020	Pf3D7_04_v3_989912	0.00010	Pf3D7_11_v3_769994	0
Pf3D7_10_v3_82375	0.00050	Pf3D7_04_v3_1032592	0	Pf3D7_11_v3_1752894	0
Pf3D7_10_v3_1402510	0.00210	Pf3D7_04_v3_1097123	0.00080	Pf3D7_11_v3_1802201	0.00180
Pf3D7_11_v3_119497	0.00110	Pf3D7_04_v3_1136990	0	Pf3D7_12_v3_83177	0
Pf3D7_11_v3_408600	0.00150	Pf3D7_05_v3_118209	0.00190	Pf3D7_12_v3_114173	0.00110
Pf3D7_13_v3_158412	0.00190	Pf3D7_05_v3_385372	0.00010	Pf3D7_12_v3_262202	0.00030
Mean F_{ST}:	0.00165	Pf3D7_06_v3_154779	0.00030	Pf3D7_12_v3_529418	0.00060
		Pf3D7_06_v3_410695	0.00040	Pf3D7_12_v3_1131606	0.00010
		Pf3D7_06_v3_593826	0	Pf3D7_12_v3_1156135	0.00010
		Pf3D7_06_v3_937752	0	Pf3D7_12_v3_1761565	0
		Pf3D7_06_v3_1184506	0.00220	Pf3D7_12_v3_2046741	0.00080
		Pf3D7_06_v3_1206498	0.01510	Pf3D7_13_v3_146745	0.00120
		Pf3D7_07_v3_420925	0.01040	Pf3D7_13_v3_210367	0.00110
		Pf3D7_07_v3_455974	0.00180	Pf3D7_13_v3_1233218	0.00080
		Pf3D7_07_v3_488471	0	Pf3D7_13_v3_1970443	0
		Pf3D7_07_v3_616673	0	Pf3D7_13_v3_2161975	0.00450
		Pf3D7_07_v3_704373	0	Pf3D7_13_v3_2573828	0
		Pf3D7_07_v3_1066647	0	Pf3D7_14_v3_1199242	0.00280
		Pf3D7_07_v3_1233830	0	Pf3D7_14_v3_1458378	0
		Pf3D7_07_v3_1256331	0	Pf3D7_14_v3_1757603	0.00200
		Pf3D7_07_v3_1268573	0.00100	Pf3D7_14_v3_1853594	0.00050
		Pf3D7_08_v3_476310	0.00650	Pf3D7_14_v3_3017749	0
		Pf3D7_08_v3_501042	0.00190	Pf3D7_14_v3_3124991	0.00210
		Pf3D7_08_v3_1100058	0.00100	Mean F_{ST}:	0.00339

Supplementary Table 11. Number of pairwise allele sharing (P_{AS}) score comparisons within each study population (study location by year) investigated for "complete monoclonal infections" dataset for 20-SNP and 75-SNP barcodes.

	**20-SNP (*N*=653)**			**75-SNP (*N*=690)**		
Study population	*N*	C	P_{AS} score (median [IQR])	*N*	C	P_{AS} score (median [IQR])
**West Africa**	**306**	**6,927**	**0.600 [0.500 - 0.650]**	**345**	**8,826**	**0.573 [0.533 - 0.613]**
**The Gambia**	**39**	**741**	**0.600 [0.500 - 0.650]**	**41**	**820**	**0.560 [0.520 - 0.600]**
Basse 2014	39	741	0.600 [0.500 - 0.650]	41	820	0.560 [0.520 - 0.600]
**Ghana**	**156**	**4,071**	**0.600 [0.500 - 0.650]**	**175**	**5,146**	**0.573 [0.533 - 0.613]**
Cape-Coast 2014	48	1,128	0.550 [0.500 - 0.650]	55	1,485	0.587 [0.547 - 0.613]
Navrongo 2010	45	990	0.550 [0.500 - 0.650]	49	1,176	0.573 [0.533 - 0.613]
Navrongo 2013	63	1,953	0.600 [0.500 - 0.650]	71	2,485	0.573 [0.533 - 0.613]
**Guinea**	**46**	**1,035**	**0.600 [0.500 - 0.650]**	**48**	**1,128**	**0.573 [0.533 - 0.613]**
Nzerekore 2011	46	1,035	0.600 [0.500 - 0.650]	48	1,128	0.573 [0.533 - 0.613]
**Mali**	**65**	**1,080**	**0.550 [0.500 - 0.650]**	**81**	**1,732**	**0.573 [0.533 - 0.613]**
Faladje 2013	40	780	0.550 [0.500 - 0.650]	52	1,326	0.573 [0.533 - 0.613]
Nioro du Sahel 2014	25	300	0.550 [0.500 - 0.650]	29	4,06	0.56 [0.52 - 0.600]
**Central Africa**	**177**	**6,022**	**0.550 [0.500 - 0.650]**	**173**	**5,858**	**0.573 [0.533 - 0.613]**
**Cameroon**	**91**	**4,095**	**0.550 [0.500 - 0.650]**	**92**	**4,186**	**0.573 [0.533 - 0.613]**
Buea 2013	91	4,095	0.550 [0.500 - 0.650]	92	4,186	0.573 [0.533 - 0.613]
**DRC**	**86**	**1,927**	**0.550 [0.500 - 0.650]**	**81**	**1,672**	**0.573 [0.533 - 0.613]**
Kinshasa 2012	54	1,431	0.550 [0.500 - 0.650]	49	1,176	0.560 [0.533 - 0.600]
Kinshasa 2013	32	496	0.600 [0.500 - 0.650]	32	496	0.587 [0.547 - 0.613]
**East Africa**	**170**	**5,398**	**0.550 [0.500 - 0.650]**	**172**	**5,582**	**0.587 [0.547 - 0.627]**
**Malawi**	**69**	**2,346**	**0.550 [0.500 - 0.650]**	**74**	**2,701**	**0.587 [0.547 - 0.627]**
Chikwawa 2011	69	2,346	0.550 [0.500 - 0.650]	74	2,701	0.587 [0.547 - 0.627]
**Tanzania**	**101**	**3,052**	**0.550 [0.500 - 0.650]**	**98**	**2,881**	**0.573 [0.547 - 0.627]**
Mkuzi-Muheza 2013	74	2,701	0.550 [0.500 - 0.650]	72	2,556	0.573 [0.547 - 0.627]
Nachingwea 2013	27	351	0.550 [0.500 - 0.650]	26	325	0.573 [0.533 - 0.613]

P_{AS} = Pairwise allele sharing score.
N = number of isolates with complete monoclonal infections (no missing data)
C = % Comparisons indicate the total proportion of pairwise comparisons between isolates within each study population.
P_{AS} values are reflected as median pairwise comparisons with interquartile ranges [IQR] within each study population.

Supplementary Table 12. Overall trend of Pairwise Allele Sharing (P_{AS}) scores when comparing within study populations using the "complete monoclonal infections" dataset.

P_{AS} Score	20-SNP (*N*=653)		75-SNP (*N*=690)	
**Total pairwise comparisons**	18,347		20,266	
**0 < 0.1**	0	0.00%	0	0.00%
**0.1 ≤ P_{AS} < 0.2**	5	0.03%	0	0.00%
**0.2 ≤ P_{AS} < 0.3**	55	0.30%	0	0.00%
**0.3 ≤ P_{AS} < 0.4**	661	3.60%	20	0.10%
**0.4 ≤ P_{AS} < 0.5**	2,898	15.79%	1,779	8.78%
**0.5 ≤ P_{AS} < 0.6**	5,889	32.10%	10,676	52.68%
**0.6 ≤ P_{AS} < 0.7**	5,752	31.35%	7,484	36.93%
**0.7 ≤ P_{AS} < 0.8**	2,527	13.77%	271	1.34%
**0.8 ≤ P_{AS} < 0.9**	505	2.75%	7	0.03%
**0.9 ≤ P_{AS} < 1.0**	33	0.18%	2	0.01%
**1.0**	22	0.12%	27	0.13%

% [*n*/*N*] *N* = total pairwise comparisons

Supplementary Table 13. Significance testing of loci on chromosome 7 for the 20-SNP barcode with variation in Navrongo, Ghana and Kinshasa, Democratic Republic of Congo (DRC) and drug resistance patterns.

	**Navrongo, Ghana** *N*=194	**Kinshasa, DRC** *N*=122
Pf3D7_07_v3_221722: A		
Sensitive	20 (10.3)	5 (4.1)
Resistant	2 (1.0)	11 (9.0)
Undetermined	1 (0.5)	0 (0)
Pf3D7_07_v3_221722: G		
Sensitive	133 (68.6)	34 (27.9)
Resistant	29 (14.9)	69 (56.6)
Undetermined	9 (4.6)	3 (2.5)
p-value	0.572	0.785
Pf3D7_07_v3_435497: A		
Sensitive	126 (64.9)	29 (23.8)
Resistant	13 (6.7)	30 (24.6)
Undetermined	6 (3.1)	1 (0.8)
Pf3D7_07_v3_435497: T		
Sensitive	27 (13.9)	10 (8.2)
Resistant	18 (9.3)	50 (41.0)
Undetermined	4 (2.1)	2 (1.6)
p-value	<0.001	<0.001
Pf3D7_07_v3_489666: C		
Sensitive	72 (37.1)	16 (13.1)
Resistant	12 (6.2)	35 (28.7)
Undetermined	6 (3.1)	1 (0.8)
Pf3D7_07_v3_489666: T		
Sensitive	81 (41.8)	23 (18.9)
Resistant	19 (9.8)	44 (36.1)
Undetermined	4 (2.1)	2 (1.6)
p-value	0.471	0.891
Pf3D7_07_v3_602559*: C		
Sensitive	60 (30.9)	17 (13.9)
Resistant	13 (6.7)	21 (17.2)
Undetermined	4 (2.1)	1 (0.8)
Pf3D7_07_v3_602559*: T		
Sensitive	57 (29.4)	18 (14.8)
Resistant	9 (4.6)	45 (36.9)
Undetermined	3 (1.5)	1 (0.8)
p-value	0.775	0.240
Pf3D7_07_v3_616459: A		
Sensitive	69 (35.6)	16 (13.1)
Resistant	13 (6.7)	38 (31.1)
Undetermined	5 (2.6)	2 (1.6)
Pf3D7_07_v3_616459: G		
Sensitive	84 (43.3)	23 (18.9)
Resistant	18 (9.3)	41 (33.6)
Undetermined	5 (2.6)	1 (0.8)
p-value	0.897	0.595

Data reflect No. (% [n/N]) of isolates from total study location population.
**NA* values for *N*=48 isolates (24.7%) at Pf3D7_07_v3_602559 in Navrongo.

Supplementary Table 14. Genotyping success from TaqMan assay in Obuasi, Ghana pre-IRS (*N*=84) and post-IRS (*N*=162).

	**Survey 1, Pre-IRS (*N*=84)**		**Survey 2, Post-IRS (*N*=162)**	
**Locus**	*n*	%	*n*	%
Pf3D7_01_v3_130339	69	82.14	152	88.10
Pf3D7_01_v3_537322	83	98.81	162	100
Pf3D7_02_v3_842805	81	96.43	162	100
Pf3D7_04_v3_276127	82	97.62	160	97.62
Pf3D7_05_v3_931606	83	98.81	161	98.81
Pf3D7_06_v3_145475	80	95.24	159	96.43
Pf3D7_06_v3_937752	79	94.05	161	98.81
Pf3D7_07_v3_221722	79	94.05	154	90.48
Pf3D7_07_v3_435497	81	96.43	161	98.81
Pf3D7_07_v3_489666	79	94.05	161	98.81
Pf3D7_07_v3_602559	70	83.33	143	77.38
Pf3D7_07_v3_616459	80	95.24	154	90.48
Pf3D7_07_v3_628386	81	96.43	157	94.05
Pf3D7_07_v3_736978	75	89.29	149	84.52
Pf3D7_07_v3_1359804	84	100	161	98.81
Pf3D7_08_v3_612596	81	96.43	156	92.86
Pf3D7_09_v3_634019	78	92.86	153	89.29
Pf3D7_10_v3_82375	77	91.67	150	85.71
Pf3D7_10_v3_1402510	81	96.43	160	97.62
Pf3D7_11_v3_119497	79	94.05	150	85.71
Pf3D7_11_v3_408600	81	96.43	159	96.43
Pf3D7_13_v3_158412	77	91.67	152	88.10
Pf3D7_13_v3_1429067	84	100	161	98.81
Pf3D7_14_v3_755731	84	100	162	100

Supplementary Table 15. The number of isolates, mixed-allele calls (MACs), major and minor alleles, minor allele frequencies (MAFs) for each of the 24 SNP loci genotyped for Survey 1 (Pre-IRS, *N*=82) and Survey 2 (Post-IRS, *N*=161). Loci highlighted in grey had a MAF ≤ 10% (0.10).

	Survey 1, Pre-IRS (*N*=82)					Survey 2, Post-IRS (*N*=161)				
Locus	*n*	MACs	Major	Minor	MAF	*n*	MACs	Major	Minor	MAF
Pf3D7_01_v3_130339	68	13	T	C	0.279	151	42	T	C	0.159
Pf3D7_01_v3_537322	81	9	A	G	0.049	161	16	A	G	0.012
Pf3D7_02_v3_842805	79	27	C	T	0.139	161	67	C	T	0.137
Pf3D7_04_v3_276127	80	14	T	C	0.088	159	35	T	C	0.044
Pf3D7_05_v3_931606	81	30	C	G	0.160	160	67	C	G	0.138
Pf3D7_06_v3_145475	79	23	C	G	0.190	158	61	C	G	0.114
Pf3D7_06_v3_937752	77	32	A	G	0.195	160	81	A	G	0.175
Pf3D7_07_v3_221722	77	15	G	A	0.013	154	41	G	A	0.019
Pf3D7_07_v3_435497	79	26	A	T	0.127	160	101	A	T	0.169
Pf3D7_07_v3_489666	77	34	T	C	0.247	160	88	T	C	0.200
Pf3D7_07_v3_602559	70	22	C	T	0.314	142	38	T	C	0.246
Pf3D7_07_v3_616459	79	32	G	A	0.215	153	64	G	A	0.235
Pf3D7_07_v3_628392	79	31	C/T	-	0	156	55	T	C	0.301
Pf3D7_07_v3_736978	73	29	A	C	0.123	149	52	A	C	0.114
Pf3D7_07_v3_1359804	82	26	C	A	0.159	160	56	C	A	0.100
Pf3D7_08_v3_612596	80	28	C	A	0.025	156	43	C	A	0.103
Pf3D7_09_v3_634019	77	40	C	T	0.117	152	98	T	C	0.125
Pf3D7_10_v3_82375	76	11	A	T	0.342	150	24	A	T	0.280
Pf3D7_10_v3_1402510	79	31	A	C	0.114	159	58	A	C	0.057
Pf3D7_11_v3_119497	77	19	G	A	0.065	150	44	G	A	0.060
Pf3D7_11_v3_408600	80	34	C	A	0.262	158	82	C	A	0.196
Pf3D7_13_v3_158412	76	34	T	C	0.145	151	86	T	C	0.146
Pf3D7_13_v3_1429067	82	2	T	-	0	160	6	T	-	0
Pf3D7_14_v3_755731	82	2	G	-	0	161	3	G	-	0

n = Number of *P. falciparum* infections; MACs= Mixed-Allele Calls
Major = Major allele per survey; Minor = Minor allele per survey; MAF = Minor Allele Frequency

References

Bei, A. K., Niang, M., Deme, A. B., Daniels, R. F., Sarr, F. D., Sokhna, C., et al. (2018). Dramatic Changes in Malaria Population Genetic Complexity in Dielmo and Ndiop, Senegal, Revealed Using Genomic Surveillance. *J. Infect. Dis.* 217, 622 627. doi: 10.1093/infdis/jix580 PMID - 29325146.

Daniels, R. F., Volkman, S. K., Milner, D. A., Mahesh, N., Neafsey, D. E., Park, D. J., et al. (2008). A general SNP-based molecular barcode for Plasmodium falciparum identification and tracking. *Malar. J.* 7, 223 11. doi: 10.1186/1475-2875-7-223 PMID - 18959790.

Nkhoma, S. C., Nair, S., Al-Saai, S., Ashley, E. A., McGready, R., Phyo, A. P., et al. (2013). Population genetic correlates of declining transmission in a human pathogen. *Mol. Ecol.* 22, 273–285. doi: 10.1111/mec.12099 PMID - 23121253.

Rice, B. L., Golden, C. D., Anjaranirina, E. J. G., Botelho, C. M., Volkman, S. K., and Hartl, D. L. (2016). Genetic evidence that the Makira region in northeastern Madagascar is a hotspot of malaria transmission. *Malar. J.* 15, 596. doi: 10.1186/s12936-016-1644-4 PMID - 27998292.
